# COPE.er Method: Combating Digital Addiction via Online Peer Support Groups

**DOI:** 10.3390/ijerph16071162

**Published:** 2019-03-31

**Authors:** Amen Alrobai, Abdullah Algashami, Huseyin Dogan, Tessa Corner, Keith Phalp, Raian Ali

**Affiliations:** 1 Department of Information Science, King Abdulaziz University, Jeddah 21589, Saudi Arabia; 2 Faculty of Science and Technology, Bournemouth University, Fern Barrow, Poole BH12 5BB, UK; aalgashami@bournemouth.ac.uk (A.A.); hdogan@bournemouth.ac.uk (H.D.); kphalp@bournemouth.ac.uk (K.P.); rali@bournemouth.ac.uk (R.A.); 3 StreetScene Addiction Recovery, Bournemouth BH1 1QA, UK; tessa@streetscene.org.uk

**Keywords:** digital addiction, online peer groups, persuasive social networks, behaviour change, persuasive systems design

## Abstract

Digital addiction (hereafter DA) denotes a problematic relationship with technology described by being compulsive, obsessive, impulsive and hasty. New research has identified cases where users’ digital behaviour shows symptoms meeting the clinical criteria of behavioural addiction. The online peer groups approach is one of the strategies to combat addictive behaviours. Unlike other behaviours, intervention and addictive usage can be on the same medium; the online space. This shared medium empowers influence techniques found in peer groups, such as self-monitoring, social surveillance, and personalised feedback, with a higher degree of interactivity, continuity and real-time communication. Social media platforms in general and online peer groups, in particular, have received little guidance as to how software design should take it into account. Careful theoretical understanding of the unique attributes and dynamics of such platforms and their intersection with gamification and persuasive techniques is needed as the ad-hoc design may cause unexpected harm. In this paper, we investigate how to facilitate the design process to ensure a systematic development of this technology. We conducted several qualitative studies including user studies and observational investigations. The primary contribution of this research is twofold: (i) a reference model for designing interactive online platforms to host peer groups and combat DA, (ii) a process model, COPE.er, inspired by the participatory design approach to building Customisable Online Persuasive Ecology by Engineering Rehabilitation strategies for different groups.

## 1. Introduction

Social software has fundamentally reshaped the way people interact. On the one hand, it provides interactive tools to build and maintain social connections and facilitate mass interactions and collaboration among individuals. On the other hand, the emergence of virtual communities and social networks and their various forms has led to changes in modern societies’ communication which can be seen negative in specific contexts and modalities of usage [1]. The increasingly notable cases in which people feel addicted to their use have also led to an increasing interest to explore this behavioural phenomenon. The patterns of use of these technological advances seem to match the criterion of Diagnostic and Statistical Manual of Mental Disorders (DSM-5) [2]. The case of online gambling is the closest example where people may immerse overly in the online space, take reckless decisions and fail to self-control their online behaviour [3].

To date, most of the recent research in DA (digital addiction) has been conducted from the social science [4] and psychological perspectives [5]. In other studies, the correlation between motives for social media use and social media addiction [6], as well as the association with gender [7] was investigated. Past literature has also shown that many studies on DA are focused on the development of usage measurement scales, e.g., [8,9,10]. We argue that software design can be a part of the DA problem and, also, support its solution. We also advocate software engineering and interactive systems design communities would need to work closely with psychology and behaviour change and empower the design of future digital technology with addiction-awareness layer and provide facilities to users to combat addictive usage patterns [11,12].

The recognition of software role and the potential of using persuasive techniques has led to a growing interest in utilising software-assisted self-regulation systems to moderate digital usage. Typically, these systems motivate users to take some responsibility to adjust their behaviour. Persuasive messages and interactive warning labels can help to initiate and maintain that change [11]. Another software-assisted system was developed to intervene with college students to reduce online usage [13]. The system offered interventions in the form of online plans based on usage which was complemented with reminder cards. The study results revealed that the intervention system efficiently reduced students’ online usage per week. In another study on smartphone addiction, the researchers proposed an intervention system to manage the usage of smartphone [14]. The system consisted of four primary functions: monitoring, data archive, data analysis, and tailored intervention based on actual usage. However, the sustainability of the change and the design process for building such software remain open issues. In addition, the study treats internet usage as being uniform and reduce the problem to a matter of time spent online which is just one aspect of DA, e.g., addictive behaviour attributes like salience and conflicts are not studied.

A study surveyed 41 smartphone intervention apps meant to help people regulate smartphone usage [15]. These apps were classified into four themes: (1) smartphone addiction diagnosing, (2) overuse intervention, (3) children use monitoring, and (4) task distraction elimination. Different persuasive techniques were used, such as self-monitoring, usage tracking and apps locking features. The study, then, highlighted that the *primary task* support dimension [16] was the dominant intervention strategy and proposed an approach to limit smartphone usage through improving self-regulation based on the Social Cognitive Theory [17], i.e., social comparison and surveillance. The approach facilitates forming groups and consists of three components: self-monitoring, goal-setting and social learning and competition.

However, most existing approaches to combat DA need clinical evidence [18]. There is also a high possibility of having adverse side-effects, such as technology dependency and anxiety about self-diagnosis [18]. Another research study found that using peer groups to mediate interventions can be harmful as it may introduce negative behaviours such as normalising the problematic behaviour and reducing its culpability due to excessive peer support [19]. Also, utilising traditional software design processes and models to build persuasive systems for behavioural change is questionable, e.g., in the notion of user requirements and its peculiarities when users can have a degree of denial and conflict in their requirements and preferences [20]. Also, the reliance on de-facto social software constructs may not be sufficient enough for designing online environment to influence behaviours for users who want to achieve specific goals and make a positive change [20]. In addition, using such systems and their features, e.g., chat and praise, to mediate behavioural change may lead to adverse consequences as they were not built for this purpose but mainly to increase openness and connectedness which is a double-edged sword if used for problematic behaviour such as DA.

In this paper, we provide a systematic approach to the design of online peer groups platforms to combat DA and minimise the potential for adverse counterproductive interactions. We highlight new challenges typically found when developing software for combatting addictive behaviours, especially when dealing with users’ requirements which have nuances and unique characteristics in this domain, e.g., using software which may cause discomfort and be in conflict to a user’s current desire to achieve a desired behaviour and style of usage in the long term. We provide engineering principles for online platforms that host peer groups and enable interventions to combat problematic behaviours associated with the use of technology. Our solution is meant for users who are willing to adjust their usage style and still at the stage of moderate addiction.

Our research studied the case of the social network as a representative example of addictive cyberspace. By social network, we mean any software-based platform for social interaction outside a business environment. We also note that addiction is a complex behaviour and usually driven by underlying issues that need to be addressed for the success of an intervention. As such, this research argues that behavioural change strategies and approaches including online peer groups will complement clinical treatment and counselling and act as an early intervention, i.e., helping addicts to start the cycle of change. However, to achieve that, the design should ensure certain pre-conditions, e.g., willingness and readiness to change, openness to shortcomings and being free from denial of reality. These can be seen as extra social requirements to ensure the success of our proposed system and to be appropriately integrated into the treatment programmes provided by professionals in treatment centres.

## 2. Background

### 2.1. Risk Factors of Digital Addiction

There are several factors that can contribute to DA. Through the review of the literature, factors were clustered into two main dimensions, individual and contextual. Mental disorders, such as attention-deficit, hyperactivity and social anxiety can be linked to DA [21]. One study identified that checking behaviours including “brief, repetitive inspection of dynamic content quickly accessible on the device” can become habitual and hence lead to some degree of addiction [22]. Another study looked at the relationships among social benefits, online social network dependency, satisfaction, and youth’s habit formation [23]. Disinhibition [24], self-disclosure [25] and hyperpersonal aspect [26] are further examples of associated behaviours.

Personality traits can influence how people interact with digital technology. Impulsive personality, which has “*tendency to respond impulsively without sufficient forethought*” [27] has been shown to have a direct link to DA [28,29]. There is also a wide range of emotions linked to DA, such as the anticipation which is an emotional motor of checking habit in that users become worried about what is going on online [30]. The anticipation is also part of escapism or the desire to change the mood state. Social network features, e.g., news and notifications and ad-hoc responses, can be argued to be using anticipation to keep users engaged. This is often framed positively as enhancing users’ experience while the potential of facilitating DA experience is often neglected.

DA relates to users’ requirements as well. People use a software product as a means to reach specific requirements such as increasing popularity and connectedness; however, while doing so, they may eventually develop a problematic usage style [12]. These requirements can be classified into three main categories: motivational, value-related and goals. The differences between these requirements and their influence on human-computer interactions were discussed by Kujala and Väänänen-Vainio-Mattila [31]. In [32], Bumgarner investigated the tacit nature of such requirements giving further types such as exhibitionism, voyeurism, conformity and social recognition. We argue in this paper that these same features can also be used to aid people to regulate their usage in a social setting. In other words, the motivations, values and goals of our particular kind of social network are to reach a usage style which is consciously regulated.

DA can also strongly relate to contextual factors including the software systems design. Designing for behavioural change, whether to make the cyberspace more engaging and immersive or to increase conscious and regulated nature of the usage, with neglecting behavioural context can lead to unintentional results based on the long-term experience. The transformation of human behaviours could be related to: (i) dispositional attribution, i.e., “The Bad Apples”, (ii) situational factors, i.e., “The Bad Barrel”, and/or (iii) the system, i.e., “The Bad Barrel-Makers” [33]. The last dimension calls for considering the design of the system that made the situation take an undesirable and unpleasant twist. In online peer groups to combat addictive behaviours, members can experience recurring episodes of relapse and denial. This may cause behavioural contagion and reinforcement of behaviour instead of correcting it. Hence, such a mechanism can be a double-sided sword to be managed in both its design and operation phases.

In terms of the software design, Young and Abreu argued that “some applications might serve as triggers for the reinforcement of continuous use [34]. This means that patients should stop navigating particular web sites or even certain applications”. The problematic usage behaviours could be triggered by external cues such as updates notifications [35]. Also, the variable discoveries by “surprise and serendipity”, such as suggesting new friends on Facebook, act as a powerful rewarding mechanism [36]. Such discoveries (aka Variable Ratio Reinforcement Schedule) provide “*variable degree of unpredictable rewards*” [34]. When these rewarding discoveries are learned and personalised, users tend to spend more time online than they initially intend to [37]. It was, also, claimed that human beings’ bodies release dopamine every time distractive updates arrive, e.g., a new likes or comments [38,39]. These updates may act as stimuli that bodies want to attain and with time people can become used to getting them to change the mood. This is an important aspect when considering the growing interest in studying the use of social media as a platform to attract collective attention and gain public recognition [40]. This, also, includes the body of research looking at different information properties (e.g., “vivid details”, interest factors, information richness and intensity) to understand their role in attracting an audience on social media [40,41].

User interface prosperities such as usability, accessibility, customisation and multitasking might also play important roles in facilitating DA. However, more studies need to be conducted to clarify the extent and significance of their influence. A study by Eyal [42] proposed the hook model which consists of four phases: trigger, action, variable reward and investment. This model was proposed to explain how companies develop habitual products. The study demonstrated that users are triggered internally or externally to perform an action, e.g., post a Facebook status. The action is performed due to an anticipated reward(s). The action phase is designed based on two usability engineering principles: ease of use and motivation. The online space designs which embrace these two principles increase the chance of users starting to take actions. These actions are then linked carefully to variable rewarding that should not be made predictable. As users invest time, money, or efforts, they are likely to be “hooked” to the software in its action-reward loop.

### 2.2. Modalities for Behavioural Change

There are different modalities for treating addictive behaviours. Modality refers to the setting of delivering treatment or a prevention approach. This includes self-help which aims at assisting individuals in obtaining behavioural interventions without attending treatment programmes [43]. It is mainly focused on enhancing individuals’ belief about their capacity, i.e., self-efficacy, to achieve their own goals [44]. Therapeutic counselling, on the other hand, is a private, often confidential and counsellor-delivered modality where individuals attend counselling sessions to express their issues, feelings and limitations. Typically, a counsellor elicits subjective aspects and descriptions of the patients’ experience while taking the role of an active and deep listener to explore their points of view and to highlight the points that need to be clarified further [34]. Support therapy focuses on providing social and emotional support. The support can be of two main categories: natural support (e.g., family and friends) and formal support (e.g., professional and communal) [45]. Peer groups can be classified as formal support if counsellors are involved, while communal if it is run as a peer-to-peer social network. It can also be operated in a blended modality where the governance and implementation of peer support is a shared responsibility between counsellors and peers. 

Support therapy can also be offered online. Online therapy is defined as “*the provision of mental health services through the Internet*” [46]. There are concerns about the full reliance on this modality and whether it shall be used in combination with face-to-face sessions, e.g., at least at the start of the therapy. One of the concerns is the impact of the patients being geographically separated from their counsellors (aka therapeutic alliance). In healthcare practices, professionals stress the need for the therapeutic relationship to increase engagement, generate hope, and ensure positive transference. This is to build objective-relationship [47] and working alliance that is conceptualised in three components: task, goal and bond [48]. In recent studies, the results suggested that online modality can also have its advantages, such as making people more comfortable and less intimidated [49]. The benefits can also be in terms of the effectiveness as the online space provide novel features of which are real-time, interactive and even immersive (e.g., virtual reality, gamified systems, role-playing, therapy networking, and online support groups). Also, it empowers self-regulation by enabling self-monitoring, behaviour tracking, and visualisation [50]. In relation to DA, the use of online support can be controversial as the online space becomes both the medium for the problem and the solution. Hence, research on the systematic design, managed interaction, and usage of online peer groups is still needed.

#### Online Peer Groups

Peer groups can be defined as a “process by which persons voluntarily come together to help each other address common problems or shared concerns” [51]. Several theoretical frameworks can help to understand the processes underpinning peer groups. This includes:

- *Self-Psychology* and its role in explaining, for example, concepts related to interpersonal conflict in social contexts (e.g., “role captivity”), the role of helping others to strengthen the identity, and how values are weighted based on the context (e.g., using the strength as an attribute to judge someone’s physical characteristics and the honesty attribute to judge the performance of a political party leader) [52].
- *Cognitive Consistency Theory* which suggests that behavioural change can motivate attitudinal change. This theory is linked to other theories such as *Self-Perception theory*, *Balance theory*, and *Cognitive dissonance*. It also highlights the role of helping others to resolve behavioural ambivalence [53].
- *Helper therapy principle* which suggests that those offering help are also benefiting from the commitment to behavioural maintenance, i.e., “*self-persuasion through persuading others*” [54]. This is also a recognised concept in Social Psychology [55]. For example, it is common to see recovered problem gamblers having their social network accounts to help others and at the same time demonstrate their new lifestyle and duration for which they are recovered which can be seen as a relapse prevention technique.
- *Social Learning Theory* which suggests that, in social contexts, some processes of the observational learning (e.g., “copying”, “internalisation”, and “role-taking”) can help to accelerate behavioural change [56].
- *Group Psychotherapy* which proposes some key factors of the help processes and dynamics when it is delivered in small groups. These factors include, for example, universality (i.e., realising that a problem is a common concern helps to alleviate isolation), altruism (i.e., the role of helping others can improve self-esteem and support the healing process), and installation of hope (i.e., increase help expectations can improve the treatment outcomes, e.g., mixing people at different stages of the rehabilitation can inspire those suffering from a higher severity and those starting the treatment) [57].

A study by Hepworth et al. [58] classified groups into two types: (i) treatment groups and (ii) task groups. Each type has distinct characteristics. In the treatment groups, the communication style follows an open style where self-disclosure discussions are expected to be high. The members’ roles evolve and are shaped through interaction over time. The progress evaluation in this type is based on meeting the treatments goals. Tasks groups, on the other hand, follow a structured communication style with low self-disclosure. Procedures are more formal, and roles are normally assigned. Achievements evaluations are based on accomplishing tasks.

Online peer groups are a type of social software that utilises certain behaviour change and persuasion mechanisms, such as social pressure through surveillance [59], to challenge negative behaviours or to reinforce positive ones [1,51]. The design of online peer groups for DA can embed features and interaction styles spanning across both treatment and task groups. The need for formality, i.e., task groups, is mainly due to the risks of reinforcing a negative behaviour or trivialising it. The need for high self-disclosure and a degree of autonomy, i.e., treatment groups, is to give a sense of ownership and commitment especially that users can be geographically distributed with little or no face-to-face contact with each other and the counsellor.

An empirical study proposed a model that views online peer groups as a tunnelling-based persuasive technique [20]. Based on the tunnelling principles articulated by Fogg [59], Alrobai et al. [20] recommended that online peer groups should have: (i) a high control over the interaction environment where the persuasion expected to occur, (ii) a carefully designed user experience where the level of uncertainties is decreased as groups progress in the treatment, (iii) a controlled or guided experience where users are walked through a pre-defined multi-stages process, and (iv) the pre-requisite that people voluntarily enter the tunnel, i.e., people in online peer groups admit having the problem and freely seeking help.

### 2.3. Theories of Behavioural Change

This section presents the main theories and models that help the understanding of the core dynamics of help provided through different modalities of behavioural awareness and change. These theories aim to explain the factors that interplay in the process of behavioural change. It can be argued that each theory and model focus on specific aspects, but they can still complement each other to provide a more holistic picture of human behaviours.

- *Theory of Planned Behaviour* [60] which is a social cognition model that emphasises the role of the intention to predict actions [61]. It is suitable to identify what to change, i.e., factors, but not to offer suggestions for change [62]. The theory constructs can be mapped to some processes of the Transtheoretical Model [63]. These processes are consciousness raising, environmental re-evaluation, dramatic relief, self-liberation. For example, self-liberation is about the belief in the ability to change, i.e., perceived behavioural control according to the theory of planned behaviour. Also, the theory can be utilised to identify which intervention strategies to use. For example, the normative influence as a persuasive principle [16] may yield better outcomes if the issue stems from erratic perception, e.g., “*no one can reduce digital usage, it is both pervasive and mandatory*”.
- *Social Cognitive Theory* [64] which is also a social cognition model that emphasises the key role of individuals’ intentions to predict actions. It shares the key principle (i.e., intention) of the Theory of Planned Behaviour but places a greater emphasis on the self-efficacy [61].
- *The Control Theory* [65] is “*a general approach to understanding the self-regulating systems*”. It requires goal(s) as a “reference value” to compare against the current rate of the behaviour. This theory is rarely used as a baseline for intervention systems for addictive behaviours due to the difficulty in setting standards [61] which stems from distorted goals (e.g., smoking improves mood) and conflicting ones (e.g., living healthy and enjoying the moment) [66]. Yet, this concept of behavioural monitoring has been widely used in self-regulating systems [61]. The use of software-assisted monitoring and feedback provides new potential for this theory for monitoring and combatting DA.
- *Transtheoretical Model* [67] which suggests that the behavioural change goes through five milestones: pre-contemplation, contemplation, preparation, action, and maintenance. It was pointed out that individuals might be trapped in one of the early stages unless the system applies planned interventions to progress them [67].
- *Health Belief Model* (HBM) [68] which has the main assumption that individuals “*must feel personally vulnerable to a health threat*”, as protective measures would be perceived necessary and, hence, potentially performed [69]. It was argued that while there is a lack of HBM-based interventions [61], the model can provide a useful understanding of DA. It was found that some constructs of the HBM (e.g., perceived benefits and barriers) are risk factors for the DA [70].
- *Goal Setting Theory* [71] which suggests that goal setting can have a positive impact on the performance. The two pillars of this theory are (i) specificity (i.e., “reference point”) in which targeting a specific goal(s) is more effective than ‘do-your-best’, and (ii) difficulty which revolves around the perceived capability to achieve the goals.

## 3. Aim, Foundations and Research Methodology

In this section, we describe our previous work, common grounds and assumptions and then elaborate on the research method which we followed to generate the results.

### 3.1. Research Aim, Background and Assumptions

This research aims to provide engineering principles for online platforms that host peer groups and intervention to combat problematic behaviours associated with the use of technology. This deals with users who are willing to adjust their usage style and still at the stage of moderate addiction.

In this research, we draw upon and extend our previous work [20]. In this prior work, we first proposed a model that highlights different transitions in group work and how the focus of the activities and tasks performed changes based on the progress through these transitions. This is to guide designers on how the platforms should operate. Secondly, we characterised the tasks performed in online peer groups in three dimensions, immediate motivators, mode of delivery, and method of delivery. Thirdly, the collected observational data helped to reveal different types of roles that can exist within small groups for behavioural change. These roles represent social status and behavioural patterns, and they are meant to inform the design process and management of the online platforms for peer groups. Finally, we revised the Honeycomb Framework [72] to expressly fit the functional requirements of online peer groups for addictive behaviours. Then, we highlighted points to consider when building online peer groups to combat addictive behaviour. In this paper, we present the rest of the results in which other substantial aspects of online peer groups are investigated. We will also propose an engineering method to guide the design of online platforms for peer support groups to combat DA.

DA has not been recognised formally as a psychological disorder yet, and we use the term mainly metaphorically. Although some research has demonstrated how DA exhibits similar symptoms to behaviour addiction [73], we emphasise that it would be hard to measure DA and judge its existence in a person due to: (i) the complexity of the issue, and (ii) the difficulty to diagnose the relation between the problematic online usage, the online space design, and online content on one hand, and the usage and more profound personal and contextual factors on the other hand. Our research is not meant to confirm or reject the existence of DA but rather to provide ways for managing what people perceive to be a problematic or addictive online usage.

According to Ng and Leong [74], there are three main stages of addiction: early, intermediate and advanced. Each stage represents a different level of self-control and distinct attitudes and behaviours. Regardless of the extent to which the object of addiction dominates decision-making processes, individuals can be guided through the levels of change according to the Transtheoretical Model [63] which articulates six levels to progress to healthier behaviour. While the online peer groups’ intervention aims at supporting individuals at all severity levels, those in the transition to addiction stage (i.e., intermediate stage) will be the main targeted audience. The reason is that tailoring the system to support those in the severe addiction stage seems to be very challenging and risky especially that our solution is meant to be run in a blended modality involving counsellors direction and, also, individuals’ autonomous self-regulation and interaction with peers.

The behavioural addictions and substance addiction have inherent similarities in terms of the symptoms and consequences. From the perspective of cognitive behavioural therapy, both types of addictions share similar diagnoses and intervention strategies [75]. This suggests that many principles, recourses, and practices in substance addiction can be adopted and applied to behavioural addiction, such as DA. Some studies, such as [76], found that Internet Addiction can be used as an important predictor for early stages of substance abuse and vice versa. This is because both addictions follow similar behavioural patterns and individuals share personality attributes [77]. Nevertheless, the variables of change, i.e., influences that could inspire individuals to change, can be different from a type of addiction to another [69].

Persuasive technology raises ethical issues, such as privacy, autonomy, social pressure, and the leaning towards designers’ intent [78]. As such, Davis in [78] argued the need for users’ involvement throughout the design process as a key aspect to uncover any ethical concerns. Then, the author recommended Value Sensitive Design (VSD) and Participatory Design (PD) as two methodological frameworks that have great potential to account for such ethical issues. In this research, we give more priorities to users’ subjective interpretations of the social phenomena they are part of and to their understandings of their own actions. This motivated our proposed method to adopt a participatory approach to systematically manage the life-cycle of the design. This is by taking an iterative and interactive approach to refine the design, reduce the number of the biased design decisions, promote communication in the development team, and, more importantly, increase adoption of the decisions and judgements made, as well as maintain interest to sustain change.

### 3.2. Research Methodology

This research is based on two observational studies to develop the first version of the reference architecture, design artefact and COPE.er method followed by a Case Study to apply them in practice and refine and consolidate them further.

#### 3.2.1. Observational Studies

We conducted two observational studies to understand peer groups including the session environment, interaction styles occurring between groups’ members and how interactions are governed. In the first study, we performed a 4-months observational study in face-to-face peer groups for treating substance and behavioural addiction. The study was performed in a rehab centre in the UK combined with interviews with an addiction counsellor to clarify the observations. The study was supported by a document analysis method using the forms and diaries utilised by patients in their daily practice. The rehabilitation centre offers inpatient residential care for patients suffering from substance addiction (including drugs and alcohol) and behavioural addiction (including gambling and sex). The rehab centre provides face-to-face group support and counselling. The management of the centre wants to extend its outreach to offer online help. Their goal is to increase treatment options by offering online help to those suffering from a problematic use of digital media as well. This is, also, to extend their help and offer online support to those in remote areas.

In the second study, we performed a 2-months observational study on an online platform for peer groups designed for treating problematic gambling and facilitated by a counsellor from a gambling recovery in the UK. The study has enabled comparing the practices in both the physical space and the cyberspace. More information about the observational study, those who were observed, and a partial analysis of the data collected can be found in [20] which discussed various design aspects of peer support groups. These aspects include group development and interaction, tasks to be performed, roles that can exist in the groups, interaction environment, and the building blocks of such platforms. These findings helped to form the basis of the proposed method in this paper. The full description of the studies can be found in [79].

Through these two observational studies and utilising our work in [80,81], which investigated the design and risk factors of persuasive intervention technology to combat DA, and our work in [1] which investigated online peer groups as a persuasive tool to support long-term behavioural change to combat DA, we developed the following outputs to be presented in the rest of the paper:

(1) A reference architecture which identifies the main components of online peer groups platforms to regulate DA (Section 5.1).
(2) A set of design artefacts to assist the design development of such platforms. The artefacts, also, includes a list of nine heuristic principles to aid stakeholders to inspect the design and to ensure optimal functionality to combat addictive behaviours (Section 5.2).
(3) A method consists of nine activities to bring focus, clear structure and the logic about the relationships between design decisions and intended functionality of online peer groups platforms. It also promotes participatory decisions making by involving end-users in the design activities (Section 5.3).

#### 3.2.2. Case Study

We conducted a case study to refine the outputs described in the previous section. The case study will aim at evaluating the proposed method in terms of the:

- *Understandability* by assessing the extent the method is easy to grasp, and whether the provided tools are useful and straightforward to understand.
- *Comprehensiveness* by assessing the extent to which the method covers different activities needed in the design process.
- *Appropriateness* by assessing the applicability of the method to the process of designing for the online space for peer groups and its ability to support the design team to incorporate various good practices.
- *Usefulness* by evaluating how the method facilitates and enhances the communication and exchange of information during the design process and how it regulates the involvement of the end-users who potentially experience problematic usage of digital media as well as the participation and role of the counsellors.

The case study approach was, also, meant to: (i) apply a holistic analysis—in the sense of multidisciplinary view—of the proposed method by recruiting participants from different disciplines, and to (ii) collect reactions and reflections on the use of the method and its supporting artefacts.

This is by applying them in practice to find out how they can be improved and whether more materials are needed to increase their quality. Also, as the research hypothesises that the proposed method will yield better results when it is utilised in a collaborative environment, e.g., focus group, we investigated how a participatory approach will contribute to the outcomes of the design process itself. This will also help to understand the dilemma of involving end-users in the design process and the concerns may arise due to the potential biased choices. The final version of these outputs, i.e., after being confirmed and refined through the case study, is presented in Section 5. In other words, Section 5 presents the final version of the artefacts after validating and refining them through the case study performed in the current paper. The material used in the case study can be found in [79].

##### Case Study Participants

The refinement of the outputs required a specific set of participants who can play different roles in the case study. The proposed engineering method is expected to be used by development teams consisting of three types of stakeholders: (i) designers who are experts in the technical side including social software design, software development and HCI, (ii) counsellors who possess the needed psychological background knowledge, and (iii) representative set of people with DA who would like to use the technology when developed.

We have used a convenience sampling via announcing the study through the mailing list of students and staff within the research group (involving both Computing and Psychology departments) and also through communicating with two addiction recovery charities in the UK. For participants who were to play the role of peer groups members, an adapted version of the alcohol use disorders screening test, which is known as the Cut Down, Annoyed, Guilty and Eye Opener (CAGE) questionnaire [82], was created to fit the properties and remit of DA and utilised as a DA screening tool. The adaptation included modifying the phrasing of the statements. For example, statement three of the original instrument which was read as follows: “Have you ever had guilty feelings about drinking?” has been rephrased to “I sometimes feel bad or guilty about my use of digital devices”. We also added two statements to enable detecting other patterns of behaviours that indicate problematic usage. These two statements read as follows: (i) “I have tried to control my use of digital devices without success”, and (ii) “I would become restless or troubled if I stop using digital devices. For end-users to be selected, the research required having two affirmative responses out of six as an inclusion criterion. The designers and counsellors were invited based on their expertise via convenience sampling. Table 1 and Table 2 provide information about the recruited participants.

The participants were given a peer group of six fictional characters of a rehab centre with DA problem, and then they were asked to create a prototype that caters for the treatment needs of those patients (i.e., group members). These six fictional members were created to act as personas that encapsulate different types of behaviours of users who might use the online peer group system. Personas are typically defined as a representation of fictional characters that are developed depending on actual users’ data to represent different types of users in the design process [83]. The given fictional six group members (i.e., personas) were developed based on the actual data obtained from the qualitative studies conducted in our prior works [1,20,81], and they mainly cover the social roles described in [20].

##### Case Study Procedure

An experienced research facilitator presented an introduction to the topic of the research, i.e., online peer groups as a motivational approach to regulating digital usage. This was followed by introducing the purpose and focus of the study. The evaluation was divided into two phases. Both phases had the same goal, but each had different tools to facilitate comparative analysis.

**Figure 1:**
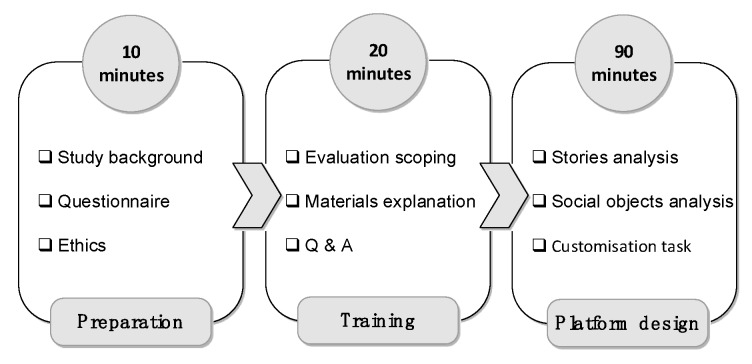
Case study first phase protocol.

**Figure 2:**
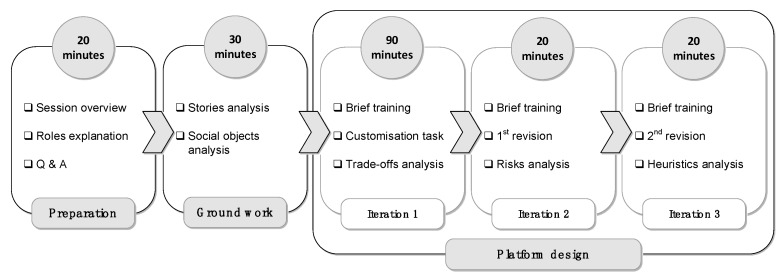
Case study second phase protocol.

- The first phase involved designing a prototype online platform for peer groups for the given case study. The goal of this phase was to investigate how the participants collaborate to design a valid and adequate platform for the given peer group without the help of a designated method. Participants during this phase were not provided with the proposed method. Then, those who played the role of designers were asked to perform the design process including the interaction with end-users and a counsellor. At this stage, the interactions were not restricted and controlled, i.e., the designers decide for themselves when and how to interact with other participates and also decide what to ask. The protocol of this phase is illustrated in Figure 1.
- The second phase had the same goal of the first phase but was conducted with the aid of our proposed method which will be introduced in Section 5. This phase was focused on consolidating the understanding of how online platforms for peer groups can be designed from different perspectives, (i.e., counsellors, end-users and designers). Also, it helped in identifying further insights to improve the method artefacts. The protocol of this phase is illustrated in Figure 2.

In both phases, the research facilitator provided the following set of guidelines to collect insights on how decisions are made with and without our proposed method:

- The designers were required to read the description of each fictional member, i.e., persona, and try to identify social roles, usage styles, general behaviours and any other aspects may have an influence on the design in terms of what features should or should not be offered to the group and how to combine and configure them.
- The COPE.er method is expected to be mainly used by designers in a design process which also involves end-users and counsellor(s), i.e., a designer-led process.
- All participants were informed in advance to the sessions about the other participants and their roles and expected contribution.
- In the first phase, i.e., without the help of the proposed method, the designers were expected to lead the process and try to involve and utilise other participants the way the designers see appropriate. In the second phase, i.e., with the help of the proposed method, guidance on how to involve and utilise them was offered.
- In both phases, the participants were provided with the same interfaces mock-ups to facilitate the discussions. The interfaces depicted an initial prototype for an online peer groups platform.
- Participants who were assigned to play the role of end-users were given the six members stories three days in advance. They were asked to read the description of each client and select one to roleplay it in both phases. They were also asked to read the descriptions of the selected members and try to simulate the effects that the addiction had on their daily life. They were also asked to be prepared for any question the designers or the counsellor may ask.

In [79], we provide detailed procedures on how the proposed method, (i.e., COPE.er) was evaluated.

#### 3.2.3. Data Analysis Approach

In this research, we adopted the qualitative content analysis approach to collect insights from the observational data. Content analysis is a qualitative-based research method concerned with producing new knowledge through a systematic analysis process of information coming from different sources, e.g., interviews, printed publications, broadcast programmes and websites [84]. It is defined by Hsieh [85] as “a research method for the subjective interpretation of the content of text data through the systematic classification process of coding and identifying themes or patterns”.

Among the three approaches to content analysis, i.e., conventional, directed and summative [85], the conventional approach was adopted. In the conventional approach, the data are analysed to derive coding categories in order to describe the phenomenon. It can be used when there is a lack of theories that explain the captured events. Based on the relationships between the articulated categories, a researcher might combine and re-organise them. With this approach, it is also essential to address relevant theories or other research findings in the discussion part of the study.

To enhance the credibility, the third author of this paper has overseen the observational studies and the analysis and provided feedback on the conduct and results throughout the research. This author has been in recovery from addiction for 30 years, worked on the creation of an organisation to provide rehabilitation from addiction, built up that organisation over the past 28 years to where it now. This organisation provides treatment for up to 54 clients at a time and is recognised by the Care Quality Commission (CQC) as going above and beyond in the provision of treatment. In [79], we present the analysis of the case study used to evaluate and consolidate our proposed method.

## 4. COPE.er: Stages and Design Principles

Part of the results in relation to the two observational studies was published in [20]. This included the social roles and the tasks performed in online peer groups, the tunnelling process and the different transitions in group work, finally a revision to the Honeycomb Framework [72]. In this paper, we will present the rest of the key findings which constitute the rest of the artefacts of our proposed method. This section starts with introducing two main set of results obtained through the observation study. The first is the primary processes identified which have been grouped into the formation phase and the acting phase processes. The second is a set of derived design principles to aid the design process of online platforms for peer support groups to combat DA.

### 4.1. Formation Phase Processes

This phase focuses on the preparatory measures to form peer groups and manage them effectively, as well as to ensure groups optimal performance.

#### 4.1.1. Assessment Processes

In the assessment stage and before permitting patients into the peer group therapy and beside the close scrutiny in relation to assessing the problematic behaviour, patients are evaluated thoroughly against certain motivational conditions including: (1) the desire to change, (2) readiness for that, (3) the stage of recovery and (4) the level of dependence. In substance addiction, part of these assessments is performed by a qualified medical doctor. Generally, patients should be joining peer groups on a voluntary basis to maximise the chance of their recovery and also to avoid disrupting others and creating negative group experience.

The assessment also covers the aspects that may influence the treatment programme, e.g., cross-addictions. For instance, a person with smartphone and social network obsessive usage could replace, or even have at the same time other kinds of addictive experiences, such as problem gambling or compulsive online shopping.

The rehab centre used to apply assessment activities iteratively for the purposes of educating patients. This was through the use of self-governed instruments such as the Assessment of Warning signs for Relapse (AWARE) scale [86] which was designed to predict the occurrence of relapse [87]. This family of assessments aims at educating individuals through guiding them to explore past experiences, i.e., warning signs and internal reactions to them, and develop self-management strategies for them. For example, when a group member selects a warning sign like “Confusion and overreaction: difficulty in managing feeling and emotions”, then with the aid of the given materials, the member can find out their more refined and subtle signs and emotions such as: (1) “*I feel that nobody would care if I tried to explain what made me unhappy*”, (2) “*I feel scared to socialise*” (3) “*I isolate myself*” (4) “*I start bringing irrelevant problem to hide the main issue which was in that case, why nobody cares?*”. As such, this assessment exercise teaches members to identify the profound and preliminary signs before the main one takes place. The goal is to help patients to avoid relapse before it takes place. This indicates that self-help and confession are essential design principles for online platforms, where patients themselves should contemplate and state the signs. This active role of patients shall have a positive impact on their ownership of their recovery process and goals.

In relation to assessing recovery, i.e., on how to distinguish whether a patient is clean or fully recovered, counsellors consider that “*experts never know but just judge that through behavioural patterns*”. Here, Here, it seems more important to have a growing amount of evidence that indicates users’ commitment to apply relapse prevention plans and strategies as well as learn effective coping skills. This would help when addictive behaviours take place outside the system environment which makes monitorability more complicated, if not impossible. In DA, this may be the evidence stage where the recovered people may take pictures of social activities and share them with the rest of the group and set up timeframes and usage targets, e.g., in terms of time, location, type and frequency, and adhere to them in a sustainable style.

We conclude that the assessment can be performed as a mixture of self-diagnosis, confession and help-seeking from the patient side and offering tools to facilitate that. The items listed in the AWARE scale proposed by Miller and Harris [86] can help the relapse prediction on online medium, e.g., having trouble in sleeping, self-pitying conversations, overreaction (e.g., through the use of Emoticons) and impulsivity, being always focused and engaged in one activity, and having no clear plans or targets.

#### 4.1.2. Matching Processes

In the treatment centre, patients with different addiction themes, e.g., gambling and substance addiction, were offered close principles and treatments. That was under the assumption that addictive behaviours share common variables in terms of initiation, maintenance and symptoms. However, there were parts of the programme which offered to target specific symptoms and behaviours. For example, anger and depression would need further therapeutic treatments, such as emotional support, anger management, changing thinking styles or even teaching some social skills. Such extra treatments can be offered in one-to-one counselling settings following the Motivational Interviewing approach. This suggests that different behavioural themes may require different treatment approaches.

Procedures for permitting patients into a group seems to ensure having a similar need(s) among all members as an essential element for better group performance [88]. However, homogeneous groups can still provide further persuasion effects. Homogeneity refers to the demographic variables (e.g., gender, age, and the ethnic group) which can help to increase receptivity to change and perhaps minimise denial. An important aspect of matching is the eventual move of users amongst different groups as they progress in the treatment, including the alternation of the participation in more than one group based on users’ needs.

In the case of relapsing, a patient must be assigned to another group. In the rehab centre, the policy stresses the need for this procedure to ensure that other patients understand that relapsing is intolerable to avoid negative reinforcement for them. Overall, patients shall recognise the importance of continuing working in their original groups as this typically provides them with more comfort and emotional support. As such, moving a relapsed member to another group can be perceived as an undesirable consequence.

To sum up, matching users to a group in the online medium may consider shared characteristics such as addiction theme, similar life experience, shared needs, demographic data, and treatment stage. This is to maximise the engagement and shared interest in the group and also avoid heterogeneity effects, such as misunderstanding and conflicts since the resolution strategies are limited in online platforms. Hence, members can easily leave if they feel discomfort or pressure.

#### 4.1.3. Preparation Processes

The rehabilitation centre divides treatments into *primary* and *secondary* classes. Primary treatment is for patients who are most vulnerable and where extra safety measures need to be applied. In secondary treatment, patients are offered psychological counselling and complementary coping skills. This may include addressing different obstacles, e.g., lack of important social skills needed in group settings. In the case of DA, this may be manifested through obstacles that may lower performance, such as lacking the experience of using certain features in online communication in the right style and failing to use a mutually accepted language when communicating with peers. The case of DA could also relate to the avoidance through living an online persona different from the actual self just to feel being accepted in some communities. The primary and secondary treatments could also be offered based on the severity of the problematic behaviours. For example, stricter measures (e.g., consent monitoring for members’ social network accounts as a part of the primary treatment protocol) are applied at the initial stages of the treatment.

Preparation is mainly concerned with preparing patients to join a group or to increase the performance after assigning them to groups. As explained in the matching stage (Section 4.1.2), this may include additional treatments such as anger management and impulse control. This is mainly for paving the way for the underlying issues to be known and then addressed and rectified when possible. Detoxification, for example, can be seen as a part of the preparation as by the end of it, addicts can pinpoint more fundamental reasons for their addictive experience.

It is important to provide descriptions of these processes from peer groups perspective. In the preparation stage, a fundamental part is to provide and apply briefing procedures in which patients are formally informed about the rehab routines, rules and guidelines for the treatment. Also, at this stage, prescribed detox plans are designed. It was observed that patients in detox can still join group sessions. Generally, detoxification is not part of the group therapy, but it can be integrated to it based on the policy of counselling service providing the treatment as well as based on the level of dependency and withdrawal symptoms of a patient. This is in line with the fact that group therapy is *a reinforcement and supportive tool* rather than being itself a primary treatment. In other words, the patient who is in the detoxification phase can join the group therapy if that is not going to introduce the risks of sabotaging the group work and even the treatment environment.

Generally, mixing senior peers with new members who might be in the detoxification stage can provide good behavioural change opportunities for both. New members can benefit from being with their senior peers who passed the detoxification stage (i.e., hope installation). It can also introduce them to the norms and good practices in the forthcoming stages of their treatment. However, in terms of designing online platforms for peer groups, this may suggest allowing controlled interactions for such users, e.g., giving them read-only permission in the group. Senior peers can also benefit from such setting as helping others can reduce and resolve behavioural ambivalence [53].

### 4.2. Acting Phase Processes

This phase focuses on the aspects related to the governance and moderation practices in peer groups. These aspects play a key role to effectively operate group work.

#### 4.2.1. Ongoing Assessment

The treatment provided in the observed groups followed a nonlinear approach and was a subject to ongoing evaluation to address the next treatment requirements and corrective measures. Ongoing assessments seem to look at both distal and proximal goals.

Distal goals are more focused on long-term progress mainly toward a balanced lifestyle as a main indicative measure. As addiction is about losing that balance, the degree of recovery can be assessed based on regaining it. In recovery performance assessment, it seems more appropriate to consider this aspect as a distal goal (i.e., advanced stage of recovery).

Proximal goals are also part of the recurrent assessment. Patients in the rehab centre decide their own specific, measurable, agreed upon, realistic and time-based (SMART) goals on a weekly basis. The selected goals can be simple ones, such as going to the gym or reading a book. An essential aspect of goals selection is that goals should encourage the performance of the healthy and balanced lifestyle tasks which addicts used to neglect or avoid. There are two main purposes of setting proximal goals. The first is to “*teach members that they need to have the right goals, learn how to decide them and to be achievable in a week*”. The second is “*to enhance their self-esteem* (through the accumulation of success)”. The activity of goals selection is done collectively where each group member can have only one goal. Typically, a therapist guides this process to ensure selecting the right goals. However, in the case of peer-led groups, i.e., where no therapist is involved, the group should help a member in selecting a goal “*because the group may know better than what an individual addict knows as the addict will be stuck in his/her own behaviours*”. There is a risk here of some members being stigmatised after repetitive failure or indeed accepting being little efficient if the process of the group is not observed.

#### 4.2.2. Membership Duration Decisions

The patients of the centre are recommended to stay in the treatment for three to six months. This indicates that behaviours of people with severe cases may need an extended period to be influenced, i.e., moving from awareness stage to adopt new healthy behaviours and maintaining them. This should inform assessment processes and feedback messages to avoid any deceptive labels related to the progress of the treatment.

The counsellor highlighted an important consideration in which behavioural change applications are not expected to maintain positive change in severe addiction cases. This is unless patients are encouraged and supported to participate in the wider community beyond their peer groups, for example, through employment opportunities to sustain positive outcomes. Therefore, such a part should be addressed in aftercare treatment where formal group sessions are less essential compared to semi-formal sessions to help patients finding and defining a focus as a purpose in their life to maintain recovery. This may suggest extending the membership to the aftercare stage, where joining group sessions is less essential, but monitoring may still be needed. This also suggests that online platforms for peer groups shall be seen a temporary platform and a part of a more holistic socio-technical process involving other stakeholders, e.g., schools for children with compulsive gaming experiences to integrate them again in class activities and after-school clubs.

#### 4.2.3. Moderation

In peer groups, some forms of interactions should be highly controlled. Private communications are generally discouraged during primary treatment. The treatment centre strives to prevent patients from staying alone without having one of the staff around during the formal sessions and even after that. In addition, deep intimacy and relation are also discouraged to avoid distraction from the main goal and creating a parallel experience. Patients in the residential rehab are “*expected to meet in the café and other public areas and not allowed to go to each other rooms*”. In conclusion, the relation between members should be moderated to prevent both isolation and deep intimacy and keep the focus on the treatment and behaviour change. On the other hand, during secondary treatment, members are encouraged to attend self-help groups and make friendship relations to help support them after treatment.

Moderation shall also be concerned about the possibility of relapse. The counsellor explained that one of the warning signs of relapse is “avoidance and defensiveness” in which an addict feels worried about others instead of self. That was also found in the materials used during the exercises performed in the sessions of the residential rehab centre. Regardless of whether avoidance is intentional or not, it is still seen as a risk factor and should be avoided and addressed by moderators.

Also, socialisation with people who are not part of the treatment may not be advised in the initial stage of the treatment, again, for the same purpose of helping members to focus on the recovery goals and treatment journey. The counsellor pointed out that this rule is mainly in primary treatment to ensure the safety of the patients as they are under the centre responsibility. The counsellor also highlighted that such policy is often applied by centres whose treatment programmes are based on the 12th steps programme of Alcoholic Anonymous (AA). The AA is a programme that provides a set of addiction recovery principles which includes admitting being powerless over addiction, examining past errors, and personal inventory of defects and successes [89]. This shows that residential treatment centres require a high level of moderation which may be difficult to replicate on the online medium. This is another reason for limiting the applicability of our proposed method to cases where people suffer moderate problematic online usage and in a status where they already admit the issue and seek voluntarily for help.

The observations also suggest that enabling peers to judge and confront each other during groups interaction can create a healthy environment. Yet, this needs the moderators’ skills to use these situations to energise group work rather than being primarily about the subject of the argument. As a cost of this openness, the moderator shall enforce a rule that no one crosses the boundaries and hurts peers’ feelings. In face to face interactions, the role of the moderator is very important to govern the interaction and elevate any negative ones that may occur. The interviewed counsellor pointed out that addicts may pay limited attention to the boundaries and norms typically observed by their society. Therefore, part of the treatment is helping them to recognise such boundaries mainly from the perspective of respecting others.

### 4.3. Design Principles for Online Peer Groups

In this section, we reflect on the above discussion and our previous work on the topic [1,20,81] and derive good practices for the design of online platforms to host peer support groups.

#### 4.3.1. The Receptive Audience Pre-Requisite

In health behaviour change, it is a fundamental prerequisite that individuals are admitting their problematic behaviour and willing to receive help. Yet, there is criticism towards emphasising self-labelling of being addicted as a requirement for treatment, i.e., the absence of this condition should not be seen as an obstacle to optimal treatment [90]. However, it seems that in rehab programmes, counsellors utilise certain principles as an assessment of motivation. In the rehab centre, the addiction counsellor stated that “*the only way to help addicts is to convince them somehow to seek help. Unless they seek help, no one can help them at all*”. This suggests that admitting the responsibility for the behaviour, both the problematic and the desired, is a crucial motivational principle. There is also what is called *dispositional attribution* in which a patient relates the responsibility to individual factors rather than external factors. Attributing the behaviour to external factors is perceived as a defensive mechanism in health behaviour change practices [91].

Miller in [90] lists four key motivational principles in the Motivational Interviewing approach: (1) “individual responsibility” to seek help, (2) placing the responsibility on “internal attribution”, (3) recognising discrepancy between addictive behaviour and personal values, goals and beliefs, i.e., “cognitive dissonance”, and (4) “increase self-esteem” via enhancing attributes that increase confidence in own abilities. These key principles are the main areas that counsellors work on to influence a behaviour in this approach. Two important motivational indicators can be elicited from the observational study and Miller’s four principles:

- Individual responsibility to seek help.
- The individual perception that the change is not beyond personal control.

However, expecting individuals to easily accept the secondary nature of the external factors and the primary nature of personal attributes is often not realistic. The assessment of this level of admittance is essential. In addition, education through the treatment programme should play an important role to help users minimising the belief on the role of external factors which may increase the probability of having long-lasting change [90].

In the case of online platforms for peer groups, it seems that a stage which deals with diagnosing the two motivational indicators shall be introduced and iteratively repeated. This is to avoid negative behavioural change which may require moving backwards into the stabilisation stage where a user needs to be re-assessed to avoid emergent withdrawal symptoms.

#### 4.3.2. Online Peer Groups as an Adaptive Ecology

**Figure 3:**
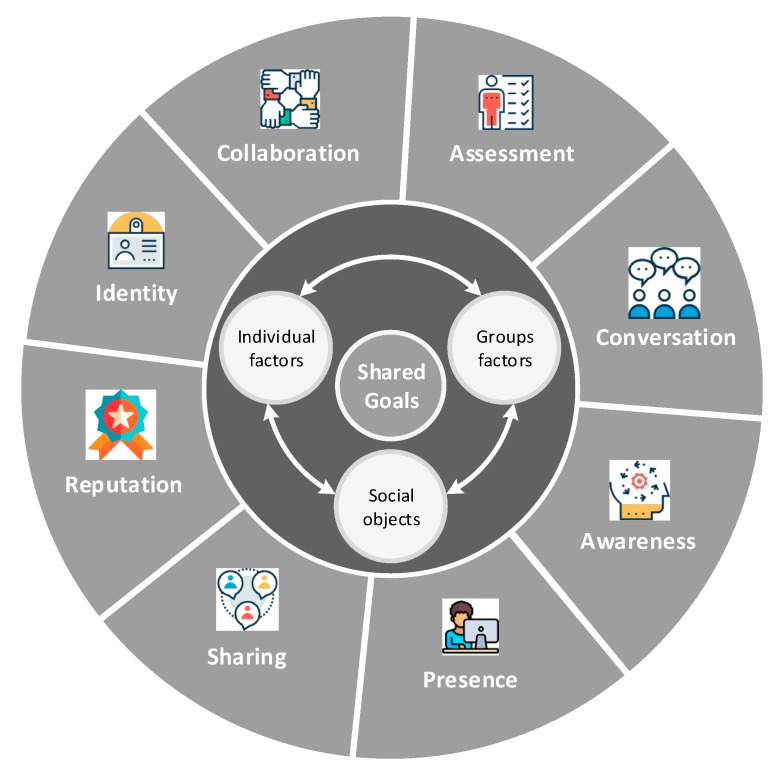
The COPE.er method building blocks.

We argue that designers of persuasive social software need to be aware of the building blocks which were introduced in the results of our initial analysis of the observation study published in [20]. In order to tailor the design features to optimise group performance, these building blocks should be configured based on four parameters which are the heart of the proposed framework shown in Figure 3. These parameters are: (i) shared goals which are excepted to boost group performance, (ii) group factors, e.g., moderation, governance aspects and group structuring [1], (iii) individual factors, e.g., personal traits, attitudes, preferences and social roles [20], and, (iv) social objects [92], e.g., topics, ideas and events.

As an illustration, social roles [20], which define patterns of behaviours that exist in the social structure of small groups, can have different influence not only on that structure and how the group is governed but also on the ecology formation (i.e., what interactive features should be offered). For example, “hope installation” as a task purpose may require some social roles, such as ‘senior’ peers to be introduced to the group in order to increase the persuasiveness of the systems. Those peers are expected to have started gaining control over their use. The system may, also, need to apply some constraints on other roles, such as limiting the sharing features for those playing the ‘relapsed’ role especially when they also play ‘dominant’ or ‘crisis’ roles. It should be noted, that some roles are not primary, but emerge from other existing ones, e.g., the ‘withdrawing’ role may be a result of having dominant users in a group. They may also emerge due to the formulation of the tasks. For example, in a very competitive task, where there might be a user taking the ‘in-detox’ role, the other members of the group may start blaming that peer for poor performance. Consequentially, the ‘scapegoating’ role emerges. Generally, these roles can help or hinder group performance. Some roles may convey positive meaning to group work, e.g., ‘helper’ and ‘sociable’, while others convey the opposite, e.g., ‘dominant’ and ‘scapegoating’. However, all these roles might be, eventually, needed for counsellors to create more effective group functioning.

These dimensions place different emphases on the interaction styles within the rehabilitation activities. In addition, they also influence what functionalities could support various tasks and purposes [20]. This suggests that the ecology of the online peer groups should be adaptive to emphasise different functional settings during the lifetime of the group. This could be achieved by applying different configurations of the honeycomb framework based on the specifications of the tasks, i.e., task purposes, qualities, and functionalities [20]. For example, some tasks and activities run on a rolling basis over a period of three months. After that, the platform should adapt to the expected changes in the individual behaviours and group performance.

In online peer groups, certain building blocks need to be emphasised based on the four parameters in the heart of the model in Figure 3. For a particular activity, the development team of the online version of peer groups, which may include, for example, therapists, software engineers, developers and stakeholders, should emphasise certain blocks but not others to boost the persuasiveness effect. For instance, it was observed in the study conducted in [20] that over a period of 6 weeks, the activities performed in the second group in the face-to-face rehab centre required a minimum opportunity and length of conversations. In online peer group platforms, if a system was highly emphasised by the conversation block in Figure 3 through the implemented features, the members’ performance would be negatively influenced, and facilitators would not be able to obtain optimum outcomes. In this particular scenario, the design of online platforms for peer groups should have the ability to reconfigure the ecology and adapt to different activities requirements which change as the treatment progress. As such, applying a static ecology approach, such as in traditional social networking services, e.g., Facebook and LinkedIn, may hinder the outcomes of the whole system and create rather a negative experience, e.g., a fake sense of achievement, lack of interest and digression.

## 5. COPE.er: A Novel Method to Design Online Peer Groups Platforms

This section starts with an introduction to the COPE.er method then continues with describing its reference architecture and supported artefacts, followed by the method workflow.

The COPE.er is a participatory method that aims to build Customisable Online Persuasive Ecology by Engineering Rehabilitation strategies for peer groups. The method provides a clear structure and logic to the relationship between design decisions and intended functionalities. It also promotes participatory decision making by involving end-users in the design activities. However, guidelines and heuristics are also provided to frame and regulate that participation and detect and handle its potential side-effects.

Customisable ecology in this context is an enabler to the online social medium that supports the adaptation of its scope, functionality, and persuasive strategies that helps to adequately cope with different group aspects, e.g., groups’ needs and progress in the rehab programme as well as governance management of groups. Media Ecology was formally introduced by Postman [93] as a way of looking into: “*how media of communication affect human perception, understanding, feeling, and value; and how our interaction with media facilitates or impedes our chances of survival. The word ecology implies the study of environments: their structure, content, and impact on people*”.

The goal of the COPE.er method is to address the challenges in designing such social networking platforms meant for combatting addictive behaviour in general and DA in particular. The COPE.er method is grounded in an extensive empirical research conducted by the authors which was itself informed by established theories in behaviour awareness and behaviour change and addictive behaviour [1,11,20,81]. The findings need to be further validated to be useful in creating effective online peer groups platforms as COPE.er is not meant to give a full specification and sharp rules on how to design the platforms and how to run the groups. Instead, it is meant to highlight phases and constituents to consider and to further customise and engineer through a participatory process. However, when evidence was obtained, we were also in the position to provide certain heuristics and best practices without a claim of completeness in that aspect.

COPE.er considers online peer groups as a specialised and domain-specific form of online social networking services. Thus, the classic Honeycomb framework proposed by in [72] would need a revision to take a decision on its fitness to this specific purpose and whether we need to refine it further or even add new blocks if needed. The COPE.er model proposes a revised version of the Honeycomb framework which matches the characteristics of peer group especially in relation to interaction, e.g., economise and optimise interactions in specific tasks and targets, and membership, e.g., screening and adherence, as well as the assessment of goals progress and its reflection on the personal and group awareness.

For example, ‘*goal progress*’ as a software feature is associated with the ‘*assessment*’, ‘*awareness*’ and *collaboration* which are new and essential building blocks that we propose through COPE.er to build online peer groups. A designer highlighted that ‘*goal progress*’ feature, for example, have a direct influence on the *social awareness*. For instance, having a peer who achieved excellent progress in a certain task(s)-oriented goals, can enhance *social awareness* and provide *collaboration* opportunities, e.g., those with low progress in these tasks can seek their peers’ help and support. Hence, it can indirectly influence the *collaboration* block. Then, they found it positive and more persuasive to make the ‘*goals progress*’ visible to all group members.

As a result, we concluded that online peers support groups should be built upon the eighth building blocks as opposed to the six blocks of the Honeycomb framework. These blocks of COPE.er are depicted in Figure 3. The new model is devised to the support the implementation of online support group platforms.

### 5.1. A Reference Architecture for Online Peer Groups

**Figure 4:**
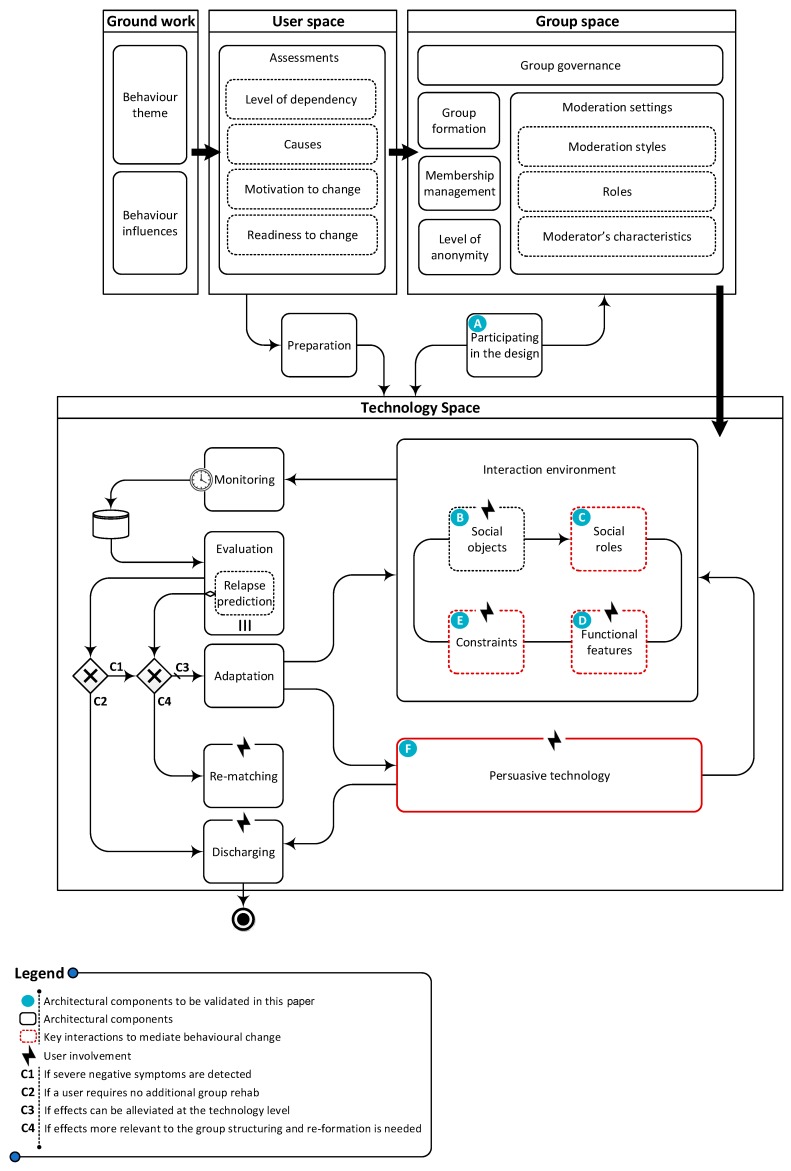
The COPE.er method reference architecture.

This section presents our reference architecture (Figure 4) which outlines the main components needed when designing online peer group platforms to regulate DA. This reference architecture has not been published yet, except the current journal. However, the components are published in our previous work which includes: (i) exploring different design aspects related to the “technology space”, mainly for persuasive techniques for E-health systems [81]; (ii) investigating online peer groups in terms of their design as a persuasive technique [1]; and (iii) exploring theoretical aspects of social software design to enable building online platforms for peer support groups as a persuasive behaviour change technique [20]. Namely, these aspects are the social roles played in the peer group and the core principles of considering peer groups as a tunnelling socio-technical persuasive paradigm.

### 5.2. COPE.er Method Artefacts

The section presents three artefacts developed based on the conducted studies of this research to facilitate the design development and support the customisation of online social platforms of peer support groups. These artefacts are (i) social objects, (ii) functional features, and (iii) design guidelines to build and customise such online platforms to combat addictive behaviours.

#### 5.2.1. Social Objects

Activities and tasks centred on social interactions need to be introduced to the online groups. The method presents these activities as social objects which help to maintain the focus on social interactions. Social objects are expected to be selected by group facilitators and negotiated with representative group members. Social objects encapsulate three aspects:

- *Purposes*: the immediate motivator(s) of the assigned task or activity, e.g., ice breaking, goals setting, hope installation, and emotional support.
- *Qualities*: the interaction orientation that mediates planned purpose(s), i.e., the mode of delivery which can include socialisation, confrontation, competition and collaboration.
- *Functionalities*: the functional activities that support achieving the planned purpose(s), i.e., the method of delivery which can include problem-solving, diaries, stories sharing, and peer pressure such as self-monitoring or surveillance.

The counsellor(s) and end-users can negotiate the treatment plan and decide what tasks and activities are suitable to be introduced to the group within the development team. Usage monitoring and surveillance are examples of core social objects for online peer group platforms that can help to manage the DA behaviours.

#### 5.2.2. Functional Features

This artefact enables the design team (including members of various backgrounds as mentioned earlier) to define the interaction environment for the online platform. It comprises a list of interactive features that can be customised and offered for different groups. The selection activity for features should consider the identified *social roles* and chosen *social objects*. The building blocks are already mapped to each feature using three colour codes; (i) dark grey, (ii) light grey, and (iii) white. These codes are described with a set of examples in Table 3. The mapping is reflected in Table 4 which has the list of the interactive features.

There are four constraints that can be applied to each feature, i.e., visibility level, usage restrictions, informational limitations and time frame. The visibility levels are embedded in the interaction environment specification table (i.e., Table 4). The designers are encouraged to create three supplementary documents to specify how the rest of the constraints should be implemented.

- *Visibility levels*: recognition and control are two opposing outcomes of visibility [94]. Visibility refers to negotiating the boundary between what can be private and public in addition to the parties who can view online social activities (e.g., posting content). Social activities are facilitated through functional features of the online platform. The visibility levels for online peer groups are; user, counsellor, specific peers, all peers, family and friends. The development team shall assess the possible combinations of these five elements and their assignment to the different features. For example, ‘*posting content*’ as a functional feature can be visible to the user only; the user and the counsellor; or all group members. Considering the group is formed for school students, the ‘*goal progress*’ as a feature may need to be visible to the user and counsellor as well as one or both parents as part of the family and friends’ visibility level.
- *Usage restrictions*: this refers to applying usage limitations to the frequency and duration of the features. The designers can assign the values; frequency (F), duration (D) or both (DF) in Table 4, and then provide more details in a separate specification document. This is illustrated by two examples shown in Table 5.
- *Informational limitations*: this refers to the information that can be accessed by a specific feature. For example, ‘*addiction scoring*’ may only consider certain applications in the calculations, e.g., games and social networks. Also, the feature may only report the type of content a user comments on, rather than the actual content, if ‘*contextualising content tracking*’ was assigned to be visible to all group members. The designers can tick (√) as shown in Table 4 and provide more details in a separate specification document. Table 6 illustrates an example.
- *Time frame*: this refers to when a feature can be enabled based on the stage of the treatment. The designers can tick (√) as shown in Table 4 and provide more details in a separate specification document. Table 7 illustrates an example. The time frame constraint utilises the transitions provided in [20].

#### 5.2.3. COPE.er Guidelines

This research proposes a set of nine heuristic principles (Table 8) that assist stakeholders to design online platforms that host peer groups for combatting addictive behaviours. Each principle includes a definition and exemplar cases. These principles are used to inspect the design by identifying problems. A development team is expected to walk through the design decisions using these principles to identify violations of the heuristics and to assess their severity.

Finally, after applying these heuristics and when problems are identified, stakeholders might decide to address them at two levels: (1) *Functional features level* by adding, removing or replacing a feature and also by modifying the level of visibility, and (2) *Group moderation level* by utilising human elements, e.g., one-to-one counselling or adopting stricter governance style.

### 5.3. COPE.er Method Activities and Workflow

**Figure 5:**
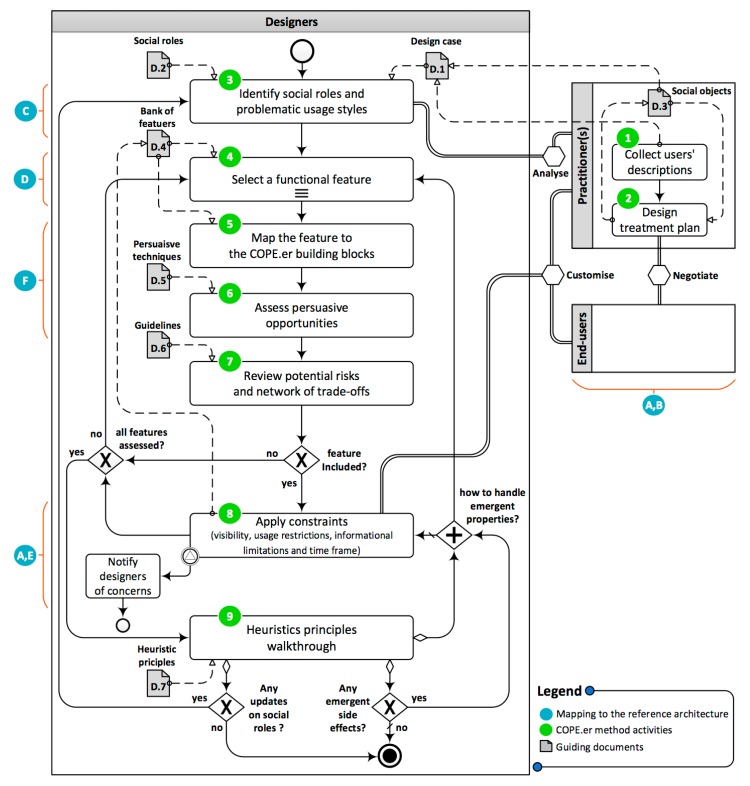
The COPE.er method workflow.

The COPE.er method follows a participatory approach to support stakeholders’ active involvement. Figure 5 illustrates the method workflow. The stakeholders are; (i) designers, (ii) counsellor(s), and (iii) end-users. The COPE.er encompasses nine activities and supported by seven documents (hereafter “D.1”, “D.2”, etc.) to guide these activities.

- *Behaviours repository (D.1)*: a document where a counsellor stores all insights about groups’ members behaviours of a given group of peers.
- *Social roles list (D.2)*: a document listing the roles exist in the social structure of small groups [20].
- *Social objects list (D.3)*: a document listing the social objects (e.g., topics for discussions, events and activities) that interactions are driven by or revolve around [20].
- *Interactive features repository (D.4)*: a bank of interactive features that can be implemented to online peer groups platforms (Table 4).
- *Persuasive techniques list (D.5)*: a document contains a list of persuasive techniques which were adopted from [16] and supported by tailored exemplar implementations for online peer groups in [79].
- *Potential risks and network of trade-offs checklist (D.6)*: the list of potential risks listed in [81].
- *Heuristics guidelines (D.7)*: a list of heuristics used to inspect online peer group platforms designs (Table 8).

*Activity 1*: A counsellor creates a document to briefly describe each member of the group. These descriptions are stored in the behaviours repository (i.e., D.1). Each member should have a separate card containing the following information:

- Member’s name or assigned pseudonym.
- General background (e.g., job, age and the date of joining the treatment centre).
- Digital usage which includes information about the usage styles including the technology being used, general motivations, and description of user feelings towards the usage.
- Counsellor’s notes which reflecting member’s social behaviour.

*Activity 2*: The counsellor decides what tasks and activities should be introduced to the group during the period of the treatment and then negotiates different aspects of the treatment with the group members. The counsellor uses the social objects list (i.e., D.3), to better describe the treatment plan to the designers. The members’ cards and the selected social objects should be added to the behaviours repository (i.e., D.1) which is the main document designers need to consider for the rest of the following activities.

*Activity 3*: The designers use the social roles list (i.e., D.2) to analyse the design case (i.e., the behaviours repository document) and identify all social roles and problematic usage styles that need to be catered for. While the designers may perform this activity alone, it is recommended to involve the counsellor. The outcomes of this activity will be reflected in the selection and customisation of the functional features of the online platform.

*Activity 4*: The designers use the interactive features repository (i.e., Table 4) to collaboratively specify the interaction environment of the online platform. In this activity, both the behaviours repository and the social roles list should be considered to enable further informed design decisions. The interactive features repository provides a bank of features and functionalities that can be implemented to the online peer group design.

*Activity 5*: The designers use the COPE.er building blocks provided in Figure 3 to decide whether to include or exclude the features being evaluated. In Table 4, all features are already mapped to the COPE.er building blocks using the colour coding provided in Table 3. However, the mapping can be revisited by the design team based on the way a feature will be implemented or combined with other features. For example, in Table 4, ‘*accomplishment*’ has a direct influence on the self-awareness and less on the social awareness. Consequently, the feature is most unlikely to create an opportunity for collaboration. However, associating ‘*badges*’ as a form of ‘*accomplishment*’ to certain tasks that require working with peers rather than self-control can indirectly influence social awareness (i.e., light grey). Hence, this can provide opportunities for collaborations.

*Activity 6*: The mapping of the features provided in Table 4 is to signal any persuasive opportunities that need to be considered. For each feature, the designers may review the persuasive techniques list (i.e., D.5). The document contains a list of persuasive techniques that are defined and explained with examples tailored for online peer groups.

*Activity 7*: The designers use the guidelines provided in the potential risks and network of trade-offs checklist (i.e., D.6) to analyse each feature and then decide how to eliminate or reduce its side-effects. The decision as to whether to include a feature or not depends on the evaluation of its impact, i.e., persuasive effect versus side-effects.

*Activity 8*: If the decision is not to include a feature, the designers assess the next item in Table 4 and then repeat the activities (5), (6) and (7). If the designers decide to include a feature, the development team (i.e., designers, a counsellor(s), and end-users) work collaboratively to customise it. The customisation focuses on applying the adequate constraints that can ultimately reduce side-effects and increase persuasion effect. The constraints encompass four types that are mentioned earlier; visibility levels, usage restrictions, informational limitations, and time frame.

*Activity 9*: This activity starts when all items in Table 4 are assessed. The designers use the nine heuristics principles in Table 8 to inspect the design of online peer groups and identify problems. Each principle has a definition and some explanatory examples.

If the problem is found in the design stage or the runtime (i.e., during actual use of the online platform, e.g., emergent side-effects), the development team address it either by revisiting activity (8) to modify the constraints or by revisiting activity (4) to check if there is a feature that may reduce the negative effect. For example, if the design features and functionalities were found to encourage private relationships, some auditing features may need to be added to provide moderators with oversight. Overall, the development team should address as many problems as possible. Then, rate the compliance to each principle on a scale of 1–5 (1 being the lowest and 5 being the highest).

Adding or removing members from a group in the runtime is likely to cause changes in the group dynamics. As such, the activity (3) should be revisited and perhaps only design decisions made on the basis of the social roles are to be reviewed. For example, there might be some features were eliminated due to the existence of specific peers in the design stage. These decisions can be re-assisted and perhaps enabled if they are found to be useful.

## 6. Method Evaluation

In this section, we present general findings of the method and highlight some concerns and design issues, method improvements and modification.

### 6.1. Governance of the Design Team Communication

At the beginning of the evaluation, there was not a clear protocol of how designers may involve other participants (counsellor and end-users) in the design process. After a discussion among themselves, the designers decided that the counsellor should be consulted only when needed, while the end-users to inquire about their preferences. In this respect, two findings were collected. Firstly, it was observed that designers overlooked the need to involve end-users to check how they would interpret some functional features. For example, one of the designers pointed out that the ‘poke’ feature is comparable to a gentle nudge “*a user can poke a peer as a wake-up call to actively participate in group work*”. Hence, they have decided to include it without asking the end-users how they would interpret it. It is a common practice over social media that *poking* can be used as a feature to get someone’s attention, e.g., flirting or saying ‘hello’. However, it is more important to inspect end-users’ interpretations of different features. Secondly, there was a lack of applying the right analysis mindset where users should not be asked what they prefer and what they want. For example, the designers asked the end-users if they would like to have some features and how their visibility should be configured. The wording of these two questions appeared to induce biased responses. End-users with addictive behaviours are prone to conscious and unconscious judgments, e.g., they might manipulate the designers. As such, the questions should be formulated in a way that encourages end-users to focus on the main purpose of the system. For example, the question of what issues or side-effects may arise if a specific feature was included is more adequate. This is to involve end-users when the designers are not sure if a certain implementation may cause harm.

Based on that, the following guidelines were derived to govern the communication among the design team members.

- The end-users and counsellor(s) are advised to interact with the designers at the step of customising the level of visibility. Also, end-users are advised to participate when there is a concern or disagreement with an assigned level of visibility.
- The counsellor can intervene when the designers overlook an aspect that might negatively affect group performance or create side effects.
- When possible side-effects are detected, five countermeasures must be analysed to select the right mitigation approach. These countermeasures are (i) modifying the level of visibility, (ii) applying some constraints, e.g., a user can post no more than five times a day, (iii) adding another feature or functionality to minimise the risk of a feature, e.g., implementing some auditing capabilities if the private communications feature is enabled, (iv) utilising direct intervention of the group moderator, e.g., through offering some tasks and activities to the group members or suggesting one-to-one counselling, and (v) providing the counsellor with some recommendations related to the group restructuring. The last countermeasure is advised based on the severity of the side-effects, e.g., affecting all peers or some of them. Restructuring the group might require re-analysing the social roles already exist in the group. For example, if a peer has addictive behaviours associated with video gaming, he/she might need to be re-assigned to another group whose interaction environment is less gamified. This is instead of removing the gaming elements from the interaction environment of the original group.

While the methodological stance of the COPE.er emphasises the domain logic as a primary focus, the involvement of the designers who had experience in some specific topics, e.g., usability, had a negative influence on the discussions in the first phase of the evaluation. For example, while the session moderator kept reminding the participants about the scope of the design, occasionally some usability issues, such as learnability, were the centre of the discussions. In the second phase, it was observed that the maintainability of the design scope had improved. Also, the designers’ communications were more focused on the core treatment requirements rather than personal preferences. However, the interaction with the end-users dropped significantly and the counsellor’s involvement was increased.

Involving the end-users without careful governance can easily inject biased decisions. Hence, controlling the wording of the questions and the situations where end-users’ involvement to be permitted seems essential aspects. Generally, while this indicates a good performance of the proposed method, such minimal participation can severely impact the ownership principle which should be promoted by the method.

In terms of the counsellor, some of the designers highlighted that the iterative consultation of the domain experts’ is very needed, others suggested the counsellor active role rather than a consultative role. Also, the number of end-users participating in the design sessions should not outweigh the number of counsellors to avoid potential bias end-users may induce.

### 6.2. Evaluating the Usefulness of the Proposed Method

In the evaluation study, we assessed how decisions are made to design the platform. The proposed method was used after the decisions were taken in the first phase to assess its usefulness to detect and correct any inadequate decisions. The findings related to the evaluation of the method usefulness are discussed below:

• *Detecting design flaws*

Generally, it was observed that some decisions seem to be based on intuitive judgements and were found to be appropriate without the need for any guidance. For example, the design team suggested that all peer-to-peer communications should be visible to the counsellor in order to avoid any adverse consequences, e.g., group clustering by which cliques may emerge.

However, other decisions that were assessed by the counsellor appeared to have adverse effects. During the first phase of the evaluation (i.e., without the aid of the proposed method), it was clear that the lack of know-how to utilise users’ stories to customise the platform resulted in inadequate design decisions that violate our derived heuristics which was not provided at this phase. For example, one of the persona’s stories read “this persona is a very kind with his peers. The persona cannot see them going emotional without trying to calm them down immediately”. The designers suggested that the system should enable the visibility of peers’ mood. Hence, the system can utilise such empathy where peers can support each other in such scenarios. Indeed, this trait is very negative in peer groups for addictive behaviours and should be discouraged immediately. Being overly emotional may cause the user to focus on others’ treatment rather than the self.

• *Elevating the risks stems from lack of understanding behavioural patterns associated with addiction*

The designers decided to reduce interaction situations when there is a high possibility of confrontational interactions between peers. For example, when a user’s performance in the treatment is not satisfactory, the system should limit the visibility of that user to the practitioner only. Matthew’s story states that “he gets intimidated when someone criticises his usage”. The designers also asked the end-users about their preference in such a scenario. In fact, such confrontational scenarios should not be avoided as long as they are objective and in a respectful tone. Healthy confrontational interactions can add positive persuasive effects to the system via *normative influence* and *peer pressure*. Generally, when it comes to common behaviours, e.g., defensiveness which is a common behaviour among addicts, the development team should not look at preferences. In other words, healthy confrontations should not be seen as an option but an essential aspect.

• *Providing a set of cognitive tools to better guide the activity of features negotiation*

When the design team wanted to include or exclude a feature, it appeared that there was a pattern of errors occur. These errors are related to overlooking the potential persuasive effect a feature may provide. For instance, the designers decided to eliminate the feature of ‘*contextualising content tracking*’ which is concerned with, for example, associating time and location to the consumed or generated content over the social media. They justified their decision by stating that the feature may trigger some privacy concerns and that may discourage them from continuing the use of the platform. They pointed out that the feature is comparable to the browsing history. Then, the researcher provided the design team with three scenarios to see how the decisions may change:

◦ Scenario (1): “Would you [the designers] include the feature, if it was at the level of what content an end-user may, for example, like, retweet or comment on, rather than the overall usage?”. The assumption here is that users’ awareness will be enhanced if they know the type of content associated with different interactions.
◦ Scenario (2): “Would you [the designers] include the feature if it was visible to the user only?”.
◦ Scenario (3): “Would you [the designers] be more inclined to think of ways to include it with a minimal side-effect, if you were reminded that this feature aims at enhancing users’ awareness?”.

After providing these three scenarios, the responses had changed to be in favour of including some eliminated features. As such, the above observation indicates the need for providing designers with extra cognitive tools, e.g., in a form of questions, to better guide this activity. Hence, the method should consistently remind designers to ask themselves (i) if there are any missing persuasive opportunities and (ii) if the decision taken may have side effects, e.g., decreasing or increasing unhealthy usage, impacting user experience, etc.

• *Bringing the persuasive techniques into the design process*

The participants were provided with a list of persuasive techniques which can be used to increase the persuasiveness of the platform. However, the participants were very unsure about how to incorporate these techniques and how they can influence the design decisions. One of the designers suggested using the list as a benchmark to see what persuasive techniques have been included in the design. As such, they decided to use it after specifying the functional features is completed. To illustrate the issue of such a decision, we provide the following design case:

The feature of declaring ‘*mood*’ is very important as it provides users with an opportunity to express their emotions. One of the fundamental aspects of treating addiction behaviours is to assess help-seekers expressing their emotions. Addiction behaviours always associated with the lack of emotional expression and quick fixes through the addiction of choice. In reference to the persuasive techniques, this feature can be mapped to the *Rehearsal principle*. In this principle, a system that is providing means with which to rehearse a behaviour can be more persuasive [16]. The feature can also be mapped to the *Social learning principle*. In this principle, users will be more motivated to perform a target behaviour if they can observe others performing the same behaviour [16].

This case led to integrating the COPE.er building blocks (Figure 3) with the bank of features (Table 4) to show which blocks a feature would have an influence upon. Generally, these findings suggest that the mapping of the features to the building blocks encourages analytical thinking, collective judgments, and helped the designers to incorporate the persuasive techniques in the analysis phase. It helped also to better understand the impact of the features and how to minimise/maximise that negative/positive impact by negotiating the parameters of the features. Parameters here refers to how a feature can be configured, e.g., in terms of the level of visibility, constraints, and feature ownership.

Finally, with regard to the method materials and the overall performance, it was observed that the method materials were very overwhelming to the participants. Also, it was apparent that the method still lacks some extra materials to help to manage the design decisions made in order to facilitate the flow of the iterations of the second phase. Hence, some extra steps are still needed to register the decisions made in each phase to inform the next one.

Further guidelines are also needed to reduce the evaluation workload. For example, the designers suggested that the user motivations should be used as a starting point in the analysis. In other words, the platform should be designed in a way to discourage the motivations for using digital media which were mentioned in the personas’ stories. For example, one of the persona’s motivations were self-presentation, passing time, and maintaining old ties. As such, the online platform should be designed against those three motivations. This suggestion can be seen as a practical approach to deal with the complexity of analysing and addressing different aspects of the personas’ stories and functional features. However, this may induce some design errors. For example, some motivations are positive and should be encouraged, e.g., relationship maintenance, and meeting new people. This indicates the need for adding guidelines related to what motivations are more relevant to the problematic usage, such as self-presentation, online romance, social comparisons and social presence, etc. and tailor the functional features in a way to discourage them.

## 7. Research Limitations

In this section, we discuss the main limitations of our research. The research has mainly targeted help-seekers. Non-help seekers may have different views about the interactive features as their perception of usefulness and attitude towards the technology can be fundamentally different, e.g., due to denial and reactance. Thus, our solution has the pre-requisite that peers are admitting the problem and voluntarily seeking help. In addition, the selection of the face-to-face and online rehab centres, where the observational studies were conducted, may have a potential influence, i.e., analysing the practices at other centres might lead to discovering additional concepts and risks. In other words, the findings can be influenced by the nature of the rehab centre, and the online forum observed and their interviewed counsellors. We mitigated this risk by basing the findings on literature and also being informed on our previous studies which involved other sources of data, e.g., coming for users through diary studies and focus groups.

Observational studies can be subject to the observer bias (e.g., to confirm hypotheses) and error (e.g., overlooking some aspect due to the lack of understanding the social context) [95]. However, the research studies conducted were exploratory in nature. Hence, there was no formal hypothesis as the purpose was to explore peer group thoroughly in order to form key hypotheses for future research. The observations and findings were verified with the experts who moderated all the observed sessions. This verification process helped to reduce human errors when collecting data. Another limitation is the heterogeneous nature of rehabilitation centres that may have different theoretical underpinnings, i.e., different centres might advocate alternative theories and approaches for treatments. The case study we conducted to refine the initial version of the method was only meant to provide a proof of concept. The method may need further enrichment and refinement in the future including testing it from other perspectives, e.g., costs and benefits analysis.

The small sample size and the selected population pose additional limitations on the generalisability of the research findings. While the research is less concerned with making generalised hypothesis statements, a larger sample size and conducting the studies in other rehabilitation centres may uncover further important perceptions.

## 8. Conclusions

We shed light on some conceptual models for understanding the design principles for social software systems. Through our rich empirical qualitative data, we showed that the existing models (e.g., [96,97]) are not sufficient to build such social platforms that have a critical focus on boosting healthy behaviours. These platforms have distinct principles that need to be considered when designing social software systems, e.g., awareness and collaboration. As such, the paper revisited the Honeycomb framework to better understanding social media audience and their engagement needs and then introduced some enhancements to cater for behavioural change requirements.

We illustrated through detailed examples that despite the design frameworks that can help to build systems for behavioural change, there is a lack of engineering methods to build online peer groups. To address this gap, the paper proposes a participatory method to facilitate the design and the customisation of online social platforms for behavioural change. Additionally, the revised building blocks are integrated into the method to better guide the identification of the persuasive opportunities in a given case design. The proposed method also provides step-by-step instructions on defining and prioritising requirements by actively involving different stakeholders as an enabler for collaborative design. The method provides the designers with the tools and guidelines, artefacts and governance protocol to effectively manage the design process and reduce potential bias that may result from end-users.

This research argues that between triggering behaviours (e.g., events, processes, etc.) and users’ reaction, there is always a choice that needs to be considered. Unfortunately, the response to a stimulus in addictive behaviours is often spontaneous and fundamentally motivational (e.g., the desire to experience thrills in gambling and online communication). So, when a technology is designed to offer and enforce users to select pre-planned choices that do not consider users’ values and actual needs, the process of addiction my start. However, the results obtained from this research suggest that the software can help to identify the right time to install a pause where users are offered a chance to rethink their usage. This is empowered by alternative choices and enforced with the aid of digital motivation techniques. The software can help to decide what alternative choices can be offered to regain control by understanding the dynamics that shaped the user experience.

Applying classical human-computer interaction principles may not be sufficient to provide designers with the right tools, principles and methods to better understand how and when to install that pause. This includes the look and feel in addition to the nature of the pause. Careful design of attention distractions can be an effective strategy to account for such spontaneous reactions in digital addiction. This strategy cannot be simply selected and applied to the software in the hope of influencing a behaviour change. The reason is that a stimulus can be enforced with other surrounding powerful elements such as hope, misconceptions, urges, and even motivations. The resulting design of social software systems (e.g., an e-health system) is likely to fail without an enhanced understanding of the complexities that are centred on digital addiction and behavioural change. Hence, this paper presented: (i) a reference model for designing interactive online platforms to host peer groups and combat DA, and (ii) a process model inspired by participatory design approach to customising such an online environment for different groups.

## Figures and Tables

**Table 1:** The background of the participants.

Participants	Role	Age	Gender	Field of Study	Years of Experience
P1	Designer	30–40	Male	Computing	13
P2	Designer	30–40	Male	Computing	8
P3	Designer	30–40	Male	Computing	5
P4	Designer	30–40	Female	Computing	5
P5	Counsellor	40–50	Male	Psychology	17
P6	End-user	20–30	Male	Computing	N/A ^1^
P7	End-user	20–30	Male	Computing	N/A ^1^
P8	End-user	20–30	Female	Computing	N/A ^1^

^1^ Not applicable as the years of experience does not apply to participants who roleplay the end-users’ role.

**Table 2:** The expert participants’ familiarity with relevant topics ^1^.

Participants	Designing for Behavioural Change					Behavioural Addiction					Human-Computer Interaction					Social Informatics					User Involvement				
	1	2	3	4	5	1	2	3	4	5	1	2	3	4	5	1	2	3	4	5	1	2	3	4	5
P1				●					●						●					●					●
P2					●					●					●					●					●
P3				●				●						●					●					●	
P4			●						●					●				●						●	
P5					●					●			●						●					●	

^1^ The questionnaire was based on 5-points Likert scale which can be interpreted as follows: (1) Very Poor (2) Poor (3) Fair (4) Good (5) Very Good.

**Table 3:** Implications on the COPE.er building blocks.

■	A feature with **great** implication on a given building block (e.g., ‘*Announcing location*’ has a **greater** implication on the *Presence* block)
■	A feature with **less or indirect** implication on a given building block (e.g., ‘*Announcing location*’ has an **indirect** implication on the *Reputation* block)
**□**	A feature with **insignificant** implication on a given building block (e.g., ‘*Announcing location*’ **does not have a significant** implication on the *Conversation* block)

**Table 4:**
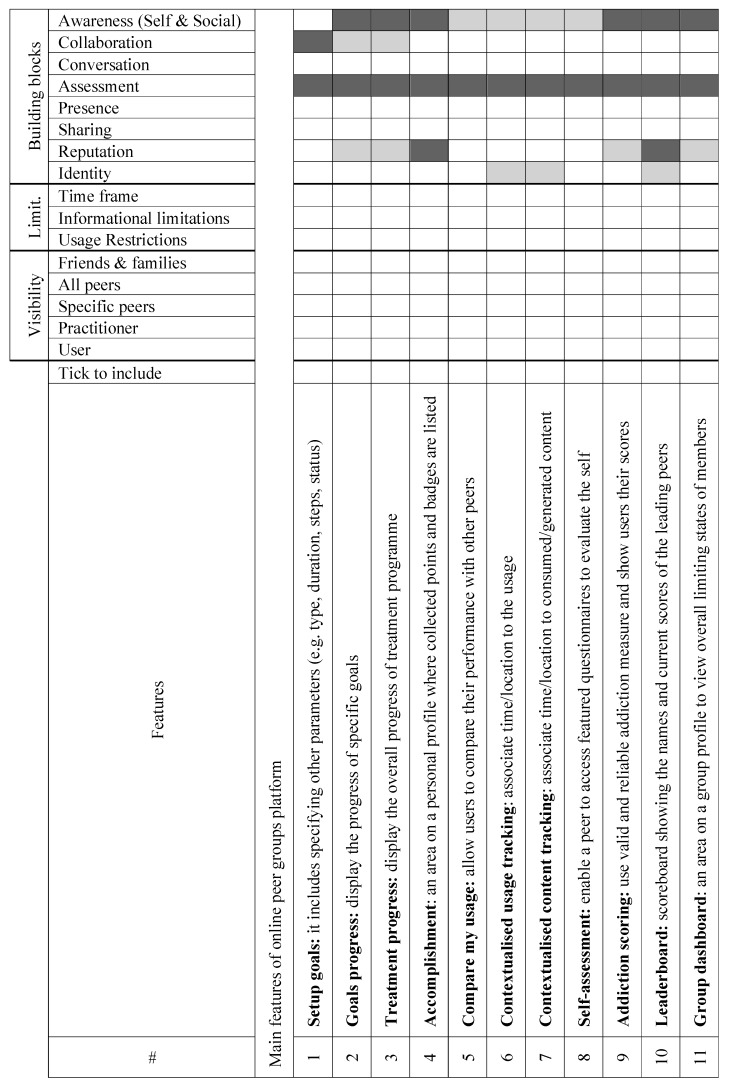

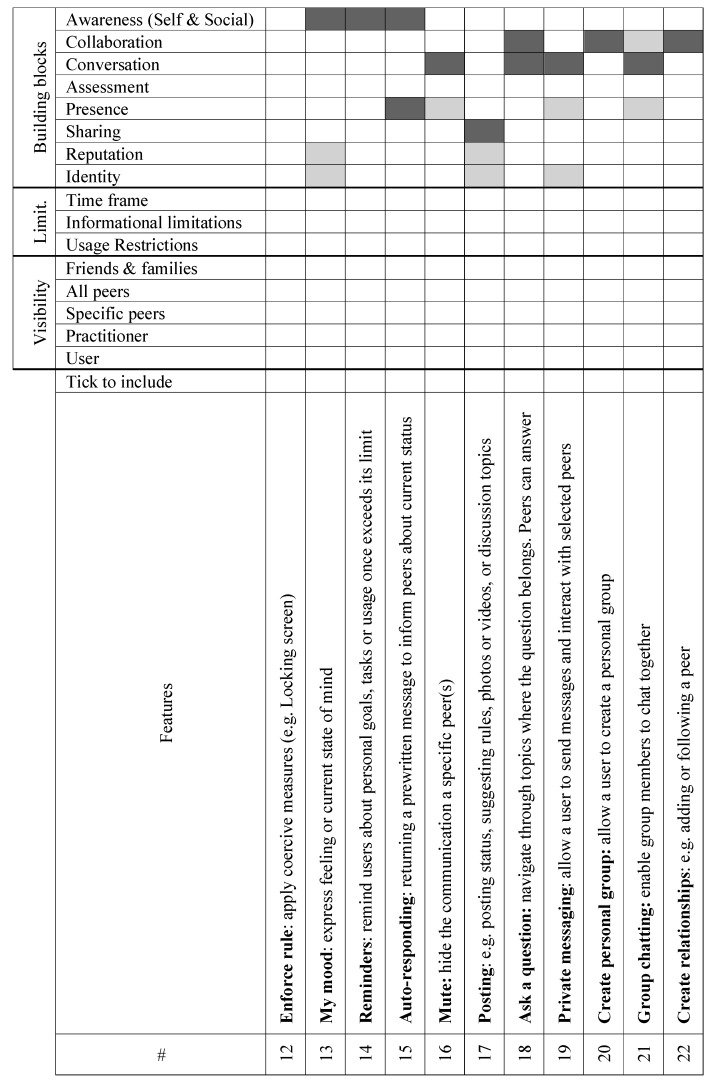

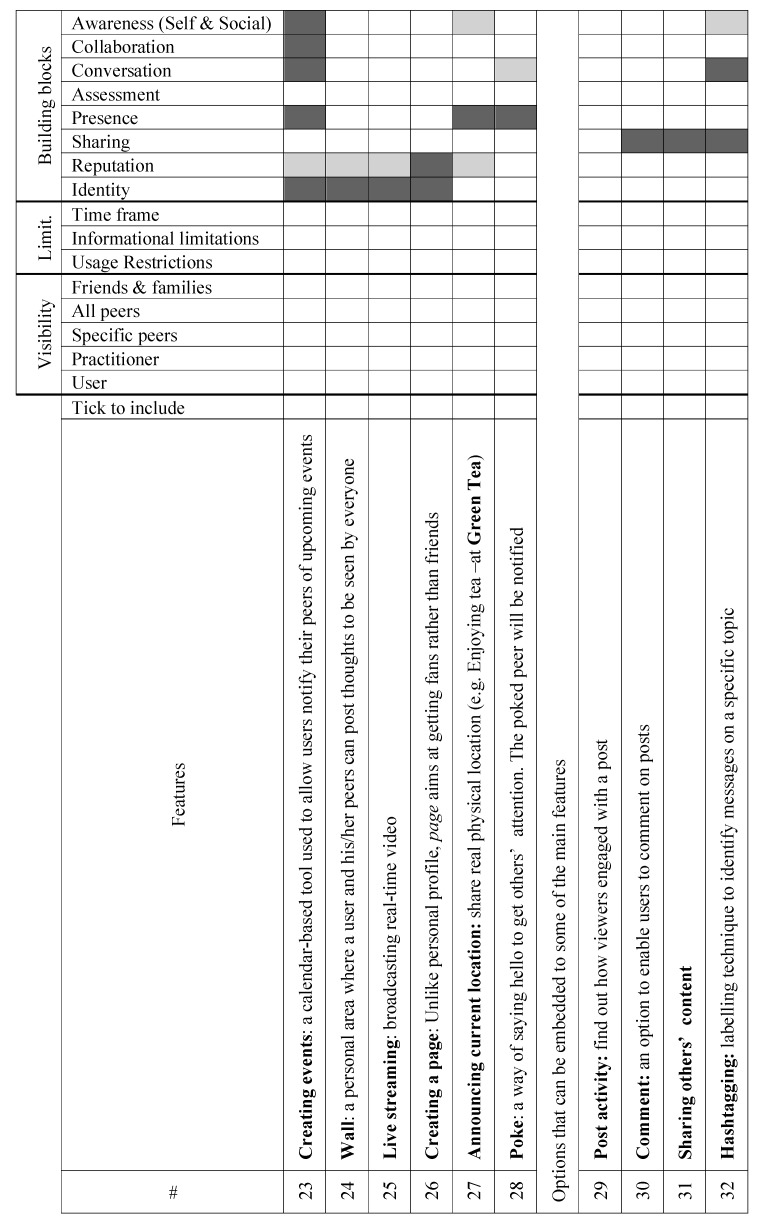

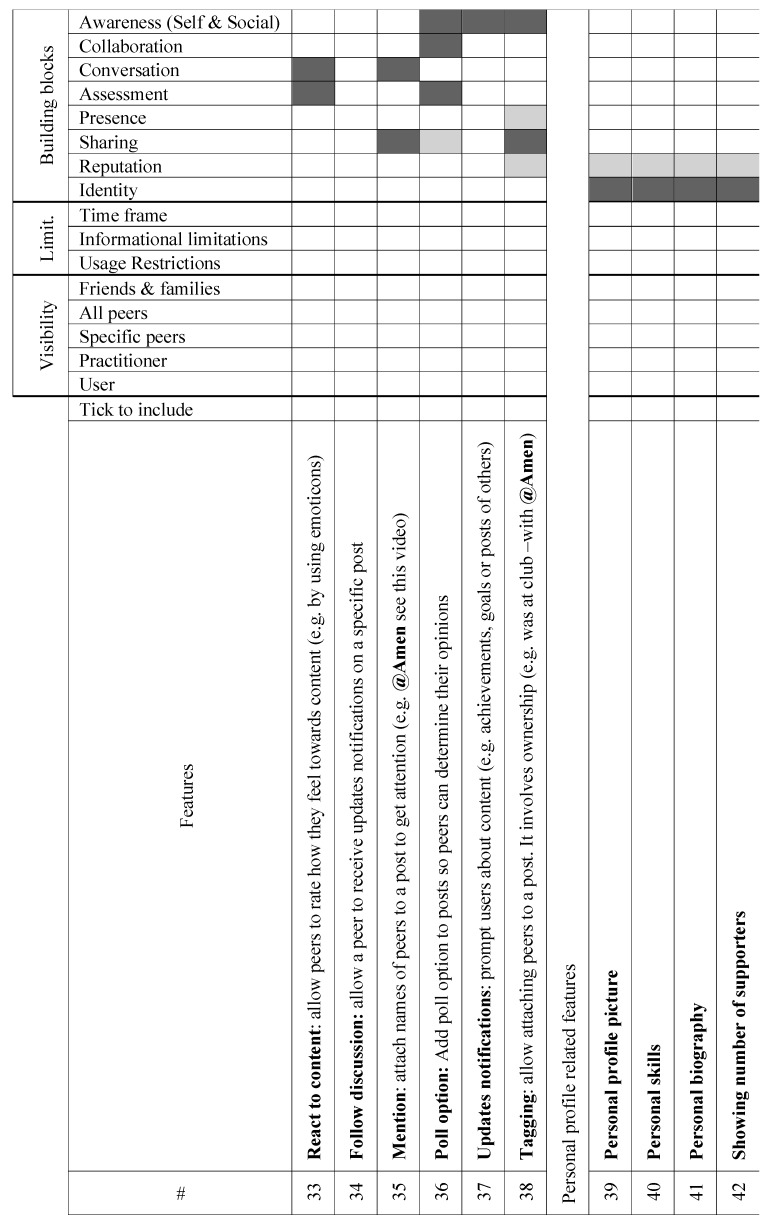
Interactive features repository.

**Table 5:** Examples for specifying frequency and duration.

Features	Frequency (F)	Duration (D)
My mood	Three times a day (7 h gap between each)	N/A
Group chatting	Only during formal group meetings	Free-floating mode during the first 30 min Round robin mode during the rest of the session

**Table 6:** An example for specifying informational limitations.

Features	Informational Limitations
Addiction scoring	Include: Facebook, Twitter and Instagram Exclude: LinkedIn, the calendar and the COPE.er app

**Table 7:** Examples for specifying time frames.

Features	Time Frame
My mood	Starts: Day 1 of the treatment programme Ends: Independency encouragement transition
Leaderboard	Starts: End of group therapy transition Ends: End of the rehabilitation programme transition

**Table 8:** Heuristic principles for inspecting online peer groups design to combat addictive behaviours.

Principles
Principle 1: Social equality rather than hierarchy Members of peer groups enjoy more democratic atmosphere where privileged positions are not explicit in group interactions. The system should boost the equity principle and give users the freedom to interact without pressure from higher-status peers. Avoid implementing features for earning social status, e.g., number of “followers” or useful comments which leads to social hierarchy.
Principle 2: Instinct to survive Confrontational communication is an inherent feature of any addiction rehab modality. However, the system should minimise triggering justification, defensiveness and denial attitude which are universal traits among addicts. Take objective stance by providing fact-based messages (e.g., usage frequency) to break through denial. Use plural pronouns “We” in messages that have negative connotations to reduce fear and to give a sense of belonging, support and empathy. The singular pronoun “I” may be used for self-judgment. Avoid sharp loss of points may trigger the feeling of “nothing is working!” or “this is not for me!”.
Principle 3: Encourage collaborative decision making Users might experience unconscious bias in selecting among alternatives that require willpower. The system should facilitate group’s collaborative decision to balance ownership and productivity. Enable users to choose visualisation format of their performance. However, goals setting is better to be selected collectively by group members.
Principle 4: Focus on the self The system should help users to focus on the self rather than walking others’ programme. Also, avoid interactions that change priorities and shift the focus away from self-improvement. The system should be a mechanism to focus on the self rather than to socialise with others. Economise surveillance. Do not emphasise peers’ evaluation to reduce self-avoidance as users more reluctant to discuss personal issues. Allow users to comment on others’ tasks if they are relevant to their group work only.
Principle 5: Prevent selective and optimised self-presentation In social situations, users often try to showcase themselves to influence others perception and to aim a specific impression. The system should discourage the motive of self-presentation and use the true-self. Profile feature in some classical social platforms (e.g., Twitter) has less emphasis on self-presentation, while others (e.g., Facebook) enable associating pictures and attitude statements to the personal profile. While groups can be provided with more freedom to feature their positive ideology, individuals should not be encouraged to do so. Avoid enabling users to keep updating their profile pictures.
Principle 6: Eliminate private relationships and subgroups Users worry about others more than the self to escape personal feeling and thoughts. The system should avoid interactions that facilitate one-to-one relationships. The system should detect users who intentionally like posts of a specific person when it is a tactic to get attention. Such interaction may lead to romance as a way of easing the pain. Avoid private communication which may lead to one-to-one relationships (e.g., add friend and poke). Users should not be enabled to self-select who they would like to see their progress, goals, badges, etc.
Principle 7: Learning before doing Users require tasks and reasonable time that match their current treatment level. The system should always start with learning-oriented tasks, goals, and actions. The system may add competition elements only in the later stages of treatment. This is to allow time for individual stabilisation, and group development, norms and cohesion. In the early stages, users may also lack adequate coping skills.
Principle 8: Encourage user self-labelling and personalisation The system should use self-labelling for behaviours that their effect remains at the individual level to increase relevance and memorability. Offer options for users to re-phrase messages in the way that describe their behaviours. For behaviours that will be seen by others, self-labelling may be manipulated to maintain reputation and self-image.
Principle 9: Emphasis dispositional attribution The system should persuade users to always relate the responsibility to individual factors rather than external factors. “Consequences” as a term stresses personal choices, while “punishment” diverts the attention away from self-responsibility. Assessment of an individual’s low-quality performance should start with addressing personal causes, while user relocation can be the last remedy. Evaluating what members add to a group rather than what the group adds to them. For example, the system may reduce the features users can use to judge qualities of the activity (e.g., suitability and difficulty) and focus on evaluating members’ performance in that activity.

## References

[1] Alrobai A., McAlaney J., Phalp K., Ali R. (2016). Online Peer Groups as a Persuasive Tool to Combat Digital Addiction. Persuasive Technology.

[2] Block J. (2008). Issues for DSM-V: Internet Addiction. Am. J. Psychiatry.

[3] Rémond J.-J., Romo L. (2018). Analysis of Gambling in the Media Related to Screens: Immersion as a Predictor of Excessive Use?. Int. J. Environ. Res. Public Health.

[4] Ryan T., Chester A., Reece J., Xenos S. (2014). The uses and abuses of Facebook: A review of Facebook addiction. J. Behav. Addict..

[5] Thomée S. (2018). Mobile Phone Use and Mental Health. A Review of the Research That Takes a Psychological Perspective on Exposure. Int. J. Environ. Res. Public Health.

[6] SZ S.S., Omar S.Z., Bolong J., Osman M.N. (2011). Facebook addiction among female university students. Revista De Administratie Publica Si Politici Sociale.

[7] Cam E., Isbulan O. (2012). A New Addiction for Teacher Candidates: Social Networks. Turk. Online J. Educ. Technol..

[8] Elphinston R.A., Noller P. (2011). Time to Face It! Facebook Intrusion and the Implications for Romantic Jealousy and Relationship Satisfaction. Cyberpsychol. Behav. Soc. Netw..

[9] Andreassen C.S., Torsheim T., Brunborg G.S. (2012). Development of a Facebook addiction scale. Psychol. Rep..

[10] Hong F.-Y., Huang D.-H., Lin H.-Y., Chiu S.-L. (2014). Analysis of the psychological traits, Facebook usage, and Facebook addiction model of Taiwanese university students. Telemat. Inform..

[11] Ali R., Jiang N., Phalp K., Muir S., McAlaney J., Fricker S.A., Schneider K. (2015). The Emerging Requirement for Digital Addiction Labels.

[12] Alrobai A., Phalp K., Ali R. (2014). Digital Addiction: A Requirements Engineering Perspective. Requir. Eng. Found. Softw. Qual..

[13] Su W., Fang X., Miller J.K., Wang Y. (2011). Internet-Based Intervention for the Treatment of Online Addiction for College Students in China: A Pilot Study of the Healthy Online Self-Helping Center. Cyberpsychol. Behav. Soc. Netw..

[14] Lee H., Ahn H., Choi S., Choi W. (2014). The SAMS: Smartphone Addiction Management System and Verification. J. Med. Syst..

[15] Ko M., Yang S., Lee J., Heizmann C., Jeong J., Lee U., Shin D., Yatani K., Song J., Chung K.-M. NUGU: A Group-based Intervention App for Improving Self-Regulation of Limiting Smartphone Use. Proceedings of the 18th ACM Conference on Computer Supported Cooperative Work & Social Computing.

[16] Torning K., Oinas-Kukkonen H. Persuasive system design: State of the art and future directions. Proceedings of the 4th International Conference on Persuasive Technology.

[17] Bandura A. (2001). Social cognitive theory: An agentic perspective. Annu. Rev. Psychol..

[18] Leigh S., Flatt S. (2015). App-based psychological interventions: Friend or foe?. Evid. Based Ment. Health.

[19] Dishion T.J., McCord J., Poulin F. (1999). When interventions harm: Peer groups and problem behavior. Am. Psychol..

[20] Alrobai A., Dogan H., Phalp K., Ali R. Building Online Platforms for Peer Support Groups as a Persuasive Behavior Change Technique. Proceedings of the International Conference on Persuasive Technology.

[21] Ko C.-H., Yen J.-Y., Chen C.-S., Yeh Y.-C., Yen C.-F. (2009). Predictive values of psychiatric symptoms for internet addiction in adolescents: A 2-year prospective study. Arch. Pediatr. Adolesc. Med..

[22] Oulasvirta A., Rattenbury T., Ma L., Raita E. (2011). Habits make smartphone use more pervasive. Pers. Ubiquit. Comput..

[23] Cheak A., Goh G., Chin T.S. (2012). Online Social Networking Addiction: Exploring Its Relationship with Social Networking Dependency and Mood Modification among Undergraduates in Malaysia. http://www.globalresearch.com.my.

[24] Suler J. (2004). The online disinhibition effect. CyberPsychol. Behav..

[25] Tamir D.I., Mitchell J.P. (2012). Disclosing information about the self is intrinsically rewarding. Proc. Natl. Acad. Sci. USA.

[26] Bellamy A., Hanewicz C. (2001). An Exploratory Analyses of the Social Nature of Internet Addiction. Electron. J. Sociol..

[27] Sternberg R.J., Grigorenko E.L. (1997). Are cognitive styles still in style?. Am. Psychol..

[28] Chwaszcz J., Lelonek-Kuleta B., Wiechetek M., Niewiadomska I., Palacz-Chrisidis A. (2018). Personality Traits, Strategies for Coping with Stress and the Level of Internet Addiction—A Study of Polish Secondary-School Students. Int. J. Environ. Res. Public Health.

[29] Sarramon C., Verdoux H., Schmitt L., Bourgeois M. (1999). Addiction and personality traits: Sensation seeking, anhedonia, impulsivity. Encephale.

[30] Griffiths M. (2000). Does Internet and computer addiction exist? Some case study evidence. CyberPsychol. Behav..

[31] Kujala S., Väänänen-Vainio-Mattila K. (2009). Value of information systems and products: Understanding the users’ perspective and values. J. Inf. Technol. Theory Appl..

[32] Bumgarner B.A. (2007). You have been poked: Exploring the uses and gratifications of Facebook among emerging adults. First Monday.

[33] Zimbardo P. (2011). The Lucifer Effect.

[34] Young K.S., de Abreu C.N. (2011). Internet Addiction: A Handbook and Guide to Evaluation and Treatment.

[35] Lee U., Lee J., Ko M., Lee C., Kim Y., Yang S., Yatani K., Gweon G., Chung K.-M., Song J. (2014). Hooked on smartphones—An exploratory study on smartphone overuse among college students. CHI.

[36] Hart J., Ridley C., Taher F., Sas C., Dix A. Exploring the facebook experience: A new approach to usability. Proceedings of the 5th Nordic conference on Human-Computer Interaction: Building Bridges.

[37] Chen J., Geyer W., Dugan C., Muller M.J., Guy I. Make new friends, but keep the old: Recommending people on social networking sites. Proceedings of the SIGCHI Conference on Human Factors in Computing Systems.

[38] Carr N. (2011). The Shallows: What the Internet Is Doing to Our Brains.

[39] Wilkinson C. Shutting out a World of Digital Distraction. http://www.telegraph.co.uk/culture/books/9522845/Shutting-out-a-world-of-digital-distraction.html.

[40] Petkova A.P., Rindova V.P., Gupta A.K. (2013). No News Is Bad News: Sensegiving Activities, Media Attention, and Venture Capital Funding of New Technology Organizations. Organ. Sci..

[41] Heath C., Tversky A. (1991). Preference and Belief: Ambiguity and Competence in Choice under Uncertainty. J. Risk Uncertain..

[42] Eyal N. (2014). Hooked: How to Build Habit-Forming Products.

[43] Lancaster T., Stead L.F., Lancaster T. (2005). Self-Help Interventions for Smoking Cessation.

[44] Watkins P.L., Clum G.A. (2007). Handbook of Self-Help Therapies.

[45] Hogan B.E., Linden W., Najarian B. (2002). Social support interventions: Do they work?. Clin. Psychol. Rev..

[46] Cook J.E., Doyle C. (2002). Working Alliance in Online Therapy as Compared to Face-to-Face Therapy—Preliminary Results. Cyberpsychol. Behav..

[47] Loewald H.W. (1960). On the Therapeutic Action of Psychoanalysis.

[48] Bordin E.S. (1979). The generalizability of the psychoanalytic concept of the working alliance. Psychother. Theory Res. Pract..

[49] Riva G., Wiederhold B.K., Cipresso P. (2017). The Psychology of Social Networking Vol.2.

[50] Barak A., Grohol J.M. (2011). Current and Future Trends in Internet-Supported Mental Health Interventions. J. Technol. Hum. Serv..

[51] Davidson L., Chinman M., Kloos B., Weingarten R., Stayner D., Tebes J.K. (2006). Peer support among individuals with severe mental illness: A review of the evidence. Clin. Psychol. Sci. Pract..

[52] Kaplan H.B. (2013). Psychosocial Stress: Trends in Theory and Research.

[53] Petri H.L., Govern J.M. (2012). Motivation: Theory, Research, and Application.

[54] Riessman F. (1965). The “Helper” Therapy Principle. Soc. Work.

[55] Leene G., Schuyt T. (2016). The Power of the Stranger: Structures and Dynamics in Social Intervention—A Theoretical Framework.

[56] Bandura A. (1997). Self-Efficacy: The Exercise of Control.

[57] Yalom I.D., Leszcz M. (2008). Theory and Practice of Group Psychotherapy.

[58] Hepworth D., Rooney R., Rooney G.D., Strom-Gottfried K., Larsen J.A. (2009). Direct Social Work Practice: Theory and Skills.

[59] Fogg B.J. (2002). Persuasive Technology: Using Computers to Change What We Think and Do (Interactive Technologies).

[60] Ajzen I. (1991). The theory of planned behavior. Organ. Behav. Hum. Decis. Process..

[61] Webb T.L., Sniehotta F.F., Michie S. (2010). Using theories of behaviour change to inform interventions for addictive behaviours. Addiction.

[62] Hardeman W., Johnston M., Johnston D., Bonetti D., Wareham N., Kinmonth A.L. (2010). Application of the Theory of Planned Behaviour in Behaviour Change Interventions: A Systematic Review. Psychol. Health.

[63] Prochaska D.J.O., Gellman M.D., Turner J.R. (2013). Transtheoretical Model of Behavior Change. Encyclopedia of Behavioral Medicine.

[64] Bandura A. (1986). Social Foundation of Thought and Action: A Social-Cognitive View.

[65] Carver C.S., Scheier M.F. (1982). Control theory: A useful conceptual framework for personality–social, clinical, and health psychology. Psychol. Bull..

[66] Sayette M.A., Griffin K.M. (2004). Self-regulatory failure and addiction. Handbook of Self-Regulation: Research, Theory, and Applications.

[67] Prochaska J.O., Velicer W.F. (1997). The Transtheoretical Model of Health Behavior Change. Am. J. Health Promot..

[68] Janz N.K., Becker M.H. (1984). The Health Belief Model: A Decade Later. Health Educ. Q..

[69] Nisbet E.K.L., Gick M.L. (2008). Can health psychology help the planet? Applying theory and models of health behaviour to environmental actions. Can. Psychol..

[70] Wang Y., Wu A.M.S., Lau J.T.F. (2016). The health belief model and number of peers with internet addiction as inter-related factors of Internet addiction among secondary school students in Hong Kong. BMC Public Health.

[71] Locke E.A., Latham G.P. (1990). A Theory of Goal Setting & Task Performance.

[72] Kietzmann J.H., Hermkens K., McCarthy I.P., Silvestre B.S. (2011). Social media? Get serious! Understanding the functional building blocks of social media. Bus. Horiz..

[73] Kuss D.J., van Rooij A.J., Shorter G.W., Griffiths M.D., van de Mheen D. (2013). Internet addiction in adolescents: Prevalence and risk factors. J. Manag. Policy Pract..

[74] Ng J., Leong E. (2009). Net Alert!: Helping Your Child Overcome Internet Addiction through Building Strong Relationships.

[75] Alavi S.S., Ferdosi M., Jannatifard F. (2012). Behavioral addiction versus substance addiction: Correspondence of psychiatric and psychological views. Int. J. Prev. Med..

[76] Fisoun V., Floros G., Siomos K., Geroukalis D., Navridis K. (2012). Internet Addiction as an Important Predictor in Early Detection of Adolescent Drug Use Experience—Implications for Research and Practice. J. Addict. Med..

[77] Lee Y.S., Han D.H., Kim S.M., Renshaw P.F. (2013). Substance abuse precedes internet addiction. Addict. Behav..

[78] Davis J. Design methods for ethical persuasive computing. Proceedings of the PERSUASIVE.

[79] Alrobai A. (2018). Engineering Social Networking to Combat Digital Addiction: The Case of Online Peer Groups. Ph.D. Thesis.

[80] Alrobai A., McAlaney J., Phalp K., Ali R. (2016). Exploring the Risk Factors of Interactive E-Health Interventions for Digital Addiction. Int. J. Sociotechnol. Knowl. Dev..

[81] Alrobai A., McAlaney J., Dogan H., Phalp K., Ali R. (2016). Exploring the Requirements and Design of Persuasive Intervention Technology to Combat Digital Addiction. HCSE/HESSD.

[82] Ewing J.A. (1984). Detecting alcoholism: The CAGE questionnaire. JAMA.

[83] Lidwell W., Holden K., Butler J. (2010). Universal Principles of Design.

[84] Lazar D.J., Feng D.J.H., Hochheiser D.H. (2010). Research Methods in Human-Computer Interaction.

[85] Hsieh H.F. (2005). Three Approaches to Qualitative Content Analysis. Qual. Health Res..

[86] Miller W.R., Harris R.J. (2000). A simple scale of Gorski’s warning signs for relapse. J. Stud. Alcohol.

[87] CASAA (2000). The AWARE Questionnaire.

[88] Toseland R.W., Rivas R.F. (2005). An Introduction to Group Work Practice.

[89] Alcoholics Anonymous the 12 Steps of AA. http://www.alcoholics-anonymous.org.uk/About-AA/The-12-Steps-of-AA.

[90] Miller W.R. (1983). Motivational Interviewing with Problem Drinkers. Behav. Cognit. Psychother..

[91] Gorski T. Denial Checklist. http://www.tgorski.com/clin_mod/dmc/denial_checklist.htm.

[92] Cetina K.K. (1997). Sociality with Objects: Social Relations in Postsocial Knowledge Societies. Theory Cult. Soc..

[93] Postman N. (1980). The reformed English curriculum. High School 1980: The Shape of the Future in American Secondary Education.

[94] Brighenti A. (2007). Visibility: A Category for the Social Sciences. Curr. Sociol..

[95] Saunders M., Lewis P., Thornhill A. (2009). Research Methods for Business Students.

[96] Charles D., McDonough S. (2014). A Participatory Design Framework for the Gamification of Rehabilitation Systems.

[97] Hoddinott P., Allan K., Avenell A., Britten J. (2010). Group Interventions to Improve Health Outcomes: A Framework for Their Design and Delivery.

